# Noncanonical NF-κB pathway driven inflammation across multiple cellular compartments identifies NIK as a therapeutic target for inflammatory bowel disease

**DOI:** 10.3389/fimmu.2026.1825442

**Published:** 2026-06-09

**Authors:** Hao Xu, Dun Li, Jie Liang, Nathan Adamson, Alexis Scherl, Luli Zou, Crystal Hu, Elaine E. Storm, Christian B. Cox, Adam Johnson, Mary E. Keir, Hua Zhang, Saiyu Hang

**Affiliations:** 1 Translational Immunology, Genentech, Inc., South San Francisco, CA, United States; 2 Immunology Discovery, Genentech, Inc., South San Francisco, CA, United States; 3 Discovery Chemistry, Genentech, Inc., South San Francisco, CA, United States; 4 Research Pathology, Genentech, Inc., South San Francisco, CA, United States; 5 Computational Biology, Genentech, Inc., South San Francisco, CA, United States; 6 Discovery Oncology, Genentech, Inc., South San Francisco, CA, United States; 7 Biochemical and Cellular Pharmacology, Genentech, Inc., South San Francisco, CA, United States

**Keywords:** inflammation, inflammatory bowel disease, mouse models and preclinical studies, NIK, non-canonical NF-kB, pharmacological inhibition

## Abstract

**Background:**

The noncanonical NF-κB pathway, driven by NF-κB-inducing kinase (NIK), plays a central role in inflammatory bowel disease (IBD) pathogenesis by regulating immune responses across epithelial, myeloid, and lymphoid compartments. Despite advances in IBD therapies, novel therapeutic options targeting NIK remain underexplored.

**Methods:**

We utilized cell-type-specific knockout models for intestinal epithelial cells, dendritic cells, as well as systemic NIK-deleted mice, to investigate the role of NIK in IBD pathogenesis. A small molecule NIK inhibitor (NIK-smi) was evaluated using pharmacokinetic/pharmacodynamic (PK/PD) modeling and its efficacy tested in preclinical colitis models, including Helicobacter hepaticus + anti-IL-10R, adoptive T cell transfer, and DSS-induced colitis. Biomarker analyses were performed using flow cytometry and multiplex cytokine detection.

**Results:**

NIK deletion in epithelial cells disrupted M-cell differentiation, reducing CCL20-mediated recruitment of CCR6-expressing Th17 cells and ILC3s. In dendritic cells, NIK activity amplified CD40-mediated innate immune responses, while in T cells, NIK skewed differentiation toward pro-inflammatory Th1 cells at the expense of regulatory T cells. Systemic NIK deletion and pharmacologic inhibition with NIK-smi attenuated inflammation in preclinical colitis models, reducing immune cell infiltration, pro-inflammatory cytokines (e.g., IL-1β, IFNγ, IL-17A, CCL5), and improving colon histopathology. PK/PD modeling revealed that a 200 mg/kg BID dosing regimen of NIK-smi provided optimal pathway inhibition and therapeutic efficacy.

**Conclusions:**

NIK is a key regulator of gut inflammation through its multi-cellular contributions in IBD pathogenesis. Pharmacologic inhibition of NIK offers a promising therapeutic strategy for IBD, with PK/PD modeling providing a translational framework to optimize dosing and efficacy.

## Introduction

The mucosal surface of the gut represents a highly intricate and dynamic multi-cellular ecosystem critical for maintaining immune homeostasis at this critical barrier site (1, 2). This system is composed of diverse cellular compartments, including intestinal epithelial cells (IECs), antigen-presenting cells (APCs) such as dendritic cells (DCs) and macrophages, as well as various lymphocytes, including T cells and B cells (3). These cellular players are in constant communication, through various stimulatory and costimulatory signaling pathways to maintain a delicate balance with the gut microbiome (4–6).

Inflammatory bowel disease (IBD), comprising Crohn’s disease (CD) and ulcerative colitis (UC), is a chronic and relapsing inflammatory disorder affecting the gastrointestinal (GI) tract (1, 4, 7). In IBD, the delicate multi-cellular homeostasis is profoundly disrupted, manifesting as impaired mucosal barrier function, increased intestinal permeability, and heightened pro-inflammatory innate and adaptive immune responses, often directed against a dysbiotic microbiome (2, 8, 9). Genetic predispositions and environmental factors contribute to this dysregulation (1, 10). Hallmarks of active IBD include significant immune cell infiltration into the intestinal mucosa, with dysregulated CD4+ T cell responses against luminal antigens playing a pivotal role in driving inflammation (11–15).

A key player in the inflammatory processes of IBD is the nuclear factor kappa B (NF-κB) signaling system, which is composed of the canonical and the noncanonical pathways (16, 17). While the canonical pathway is primarily known for its role in initiating and amplifying inflammatory responses (16), the noncanonical NF-κB pathway is critical for immune organogenesis, lymphocyte trafficking, and the priming of adaptive immunity (17). This noncanonical pathway is activated by a subset of TNF superfamily receptors, including lymphotoxin-beta receptor (LTβR), B cell activating factor receptor (BAFF-R), CD40, RANK, OX40, and Fn14, which orchestrate signaling across diverse cellular compartments (17). Importantly, genetic studies have linked NFKB2, a central gene of the noncanonical pathway, to IBD susceptibility loci (5, 18).

At the core of the noncanonical NF-κB pathway is the NF-κB-inducing kinase (NIK), which is indispensable for pathway activation. NIK kinase activity drives the proteolytic processing of p100 into p52, enabling the nuclear translocation of RelB:p52 heterodimers and subsequent regulation of homeostatic and pathogenic cytokines and chemokines (19, 20). NIK is expressed in a wide array of cell types, including IECs (21), DCs (22, 23), T and B cells (24–26). Notably, studies have demonstrated that the noncanonical pathway is upregulated in IBD, as evidenced by elevated p52-to-p100 ratios in patient samples (27). Furthermore, IBD patients resistant to anti-TNF treatment show increased expression of the NIK gene and its downstream components, including CXCL12, CXCL13 and CXCR4 (28).

Despite these observations, the cell type-specific functions of NIK in the intestinal epithelium, myeloid cells, and T cells during chronic intestinal inflammation remain incompletely defined. In particular, how NIK-dependent transcriptional programs in these distinct compartments converge to shape IBD-associated immune responses, and how NIK integrates signals from multiple TNF superfamily receptors (e.g., RANK, CD40, OX40) *in vivo*, is not well understood. Moreover, the extent to which systemic or pharmacologic NIK inhibition can ameliorate complex, microbiota-driven colitis while preserving essential host defense and tissue homeostasis has not been comprehensively investigated.

In this study, we sought to comprehensively investigate the role of NIK and the noncanonical NF-κB pathway within distinct cellular compartments, including IECs, DCs, and T cells. Using multiple pathway-driven preclinical models of IBD, we demonstrated that NIK acts as a key modulator of the inflammatory response. Our findings underscore NIK’s central role within the gut’s multi-cellular landscape and highlight its potential as a therapeutic target for IBD.

## Material and methods

### Mice

C57BL/6N mice were obtained from Charles River Laboratories or Envigo RMS LLC. Rag2^-/-^ mice were obtained from Taconic Biosciences. Villin^CRE-ERT2^ Nik^flox/flox^, Rag1^-/-^ CD11c^CRE^ Nik^flox/flox^, Rosa26^CRE-ERT2^ Nik^flox/flox^ and their littermate control mice are generated and bred at Genentech. All animal activities were conducted in accordance with the Guide for the Care and Use of Laboratory Animals (National Research Council 2011), and all animals were maintained in a facility accredited by AAALAC International. All animal activities in this research study were reviewed and approved by the Genentech Institutional Animal Care and Use Committee (IACUC) and were performed in accordance with all institutional program requirements. Mice were housed in individually ventilated cages within animal rooms maintained on a 14:10-hour, light:dark cycle. Animal rooms were temperature and humidity-controlled, between 68 to 79°F (20.0 to 26.1 °C) and 30 to 70% humidity respectively, with 10 to 15 room air exchanges per hour.

### Citrobacter rodentium infection

*Citrobacter rodentium* strain DBS100 (ATCC, #51459) was revived from frozen stocks by overnight culturing in LB broth at 37 °C. The overnight culture was sub-cultured the following day into fresh LB broth to reach mid-log phase growth. C57BL/6N mice were inoculated by oral gavage with 2 x 10^9^ colony-forming units (CFU). Body weight was monitored daily. On Day 10, mice were euthanized and colon tissues were collected. The tissues were cleaned of fat and immediately placed in ice-cold PBS for subsequent analyses. Colon density was calculated as colon weight divided by colon length (mg/cm) and used as an integrated measure of tissue edema, inflammatory cell infiltration, and wall thickening.

### anti-CD40 induced colitis

Rag1^-/-^ CD11c^CRE^ Nik^flox/flox^ mice and their littermates controls, aged 8~10 weeks old, were administered an agonist anti-CD40 antibody (Clone FGK4.5, made at Genentech) at 10mg/kg via tail vein (i.v.) on Day 0. Mice were daily monitored for body weight and terminated on Day 7. At euthanasia, colon tissues were measured and collected for subsequent pathological or flow cytometric analysis. For NIK-smi related studies, Rag1^-/-^ mice aged 8~9 weeks old were used. NIK-smi or vehicle control was administered twice daily (BID) at 200mg/kg via oral gavage (p.o.), starting from Day 0. Serum samples were harvested through retro-orbital bleeding for mass spectrometry-based PK/PD analysis, while colon tissues were harvested for cytokine analysis using the Luminex platform.

### T cell transfer colitis

Rag1^-/-^ mice aged 8~9 weeks old from Taconic Biosciences were used as recipients after stabilizing at Genentech vivarium for 3~4 weeks. For NIK knockout-related studies, 0.5 x 10^6^ naive CD4 T cells (defined as CD4^+^, TCRb^+^, CD25^-^, CD45RB^Hi^ and purified with FACS-based sorting) isolated from the spleens of Rosa26^CRE-ERT2^ Nik^flox/flox^ mice or their littermate controls were administered to recipients on Day 0 via intraperitoneal (i.p.) injections. To induce NIK depletion in adoptively transferred CD4 T cells, tamoxifen (Sigma, #T5648) was dissolved in sterile sunflower oil (Sigma, #S5007) and dosed to recipient mice via intraperitoneal (i.p.) injections at 80mg/kg once daily from Day 1 to Day 3. On Day 21, colon tissues were measured and collected for flow cytometric analysis (proximal half) and cytokine analysis (distal half) using the Luminex platform. For NIK-smi related studies, Wild-type C57BL/6N mice aged 6~7 weeks old were used for isolating donor CD4 T cells. NIK-smi or vehicle control were given to mice twice daily (BID) at 200mg/kg via oral gavage (p.o.) starting from Day 0 post adoptive transfer. On Day 14, colon tissues were measured and collected for flow cytometric analysis (proximal half) and cytokine analysis (distal half).

### *Helicobacter hepaticus* and anti-IL10R induced colitis

Rosa26^CRE-ERT2^ Nik^flox/flox^ mice and their littermate controls, aged 6~8 weeks old, were utilized in Fig5-related studies. On Day 0, animals were treated via oral gavage (p.o.) with 4.5 x 10^8^ CFU of *Helicobacter hepaticus* (ATCC, #51449), and via intraperitoneal (i.p.) injection with 1mg anti-CD210 antibody (Clone 1B1.3a, made at Genentech). On Day 1, a second dose of *Helicobacter hepaticus* was administered to ensure colonization. Anti-CD210 treatment was later maintained (1mg/mouse/week) via i.p. injections. To induce NIK depletion, tamoxifen (Sigma, #T5648) was dissolved in sterile sunflower oil (Sigma, #S5007) and dosed to mice via subcutaneous injection (s.c.) at 80mg/kg once daily from Day 0 to Day 5. On Day 14, fecal samples were collected for LCN2 measurement according to manufacturer’s protocol (R&D Systems, #DY1857). On Day 28, cecum and colon tissues were measured and collected for cytokine analysis using the Luminex platform. For NIK-smi related studies, Wild-type C57BL/6N mice aged 6~7 weeks old were used for disease induction. NIK-smi or vehicle control were given to mice twice daily (BID) at 200mg/kg via oral gavage (p.o.) starting from Day 0. On Day 14, cecum and colon tissues were measured and collected for cytokine analysis using the Luminex platform.

### DSS induced colitis

Wild-type C57BL/6N mice aged 8~9 weeks old were used. Starting from Day 0, 1.25% (w/v) colitis-grade DSS (MP Biomedicals) was added to the drinking water for 7 days. On Day 8, mice were re-grouped by their body weight loss, and started to receive NIK-smi or vehicle control twice daily (BID) at 200mg/kg via oral gavage (p.o.). On Day 14, colon tissues were measured and collected for cytokine analysis using the Luminex platform.

### GP2 immunohistochemistry and CCL20 *in situ* hybridization on human intestinal mucosal biopsies

Formalin-fixed, paraffin-embedded (FFPE) human intestinal mucosal biopsies and surgical resections were obtained through Genentech’s internal human tissue procurement program from established commercial vendors that conform to all relevant legal requirements for the sale and distribution of human-derived specimens. As part of our standard sample procurement workflow, for each procured sample, informed consent is confirmed, corresponding clinical pathology reports are obtained to confirm patient diagnosis (Crohn’s disease, ulcerative colitis, or non-IBD control), and anatomical site (ileum or colon) is confirmed both by vendor clinical data and manual pathologist review. Only samples with appropriate documentation, consented unrestricted usage, and pathologic tissue identity confirmation were included in this study.

IHC and ISH were performed on formalin-fixed, paraffin embedded human intestinal mucosal biopsies. For both assays, 4 micron sections were sectioned onto Superfrost plus glass slides and subjected to deparaffinization. For IHC, staining was performed using a Ventana Discovery XT autostainer per standard protocol. In brief, deparaffinization was followed by antigen retrieval with Ventana CC1 standard solution. Incubation with primary antibody was then performed (Invitrogen polyclonal GP-2 antibody, catalog# PA5-53471, 0.025ug/mL, 60 minutes at 37 °C), followed by incubation with Ventana Rabbit OmniMap secondary antibody, and detection with Ventana DAB chromogen and hematoxylin counterstain. For ISH, hybridization was performed using the RNAscope 2.5 LSx kit system (Advanced Cell Diagnostics) on a Leica Biosystems BOND RX autostainer per standard protocol. In brief, deparaffinization was followed by protease digestion (Protease III), followed by hybridization with the probe set (Hs-CCL20 nt20-811, NM_001130046.1, catalog#409618, 15 molar concentration, 120 minutes at 42C), followed by detection with Fast Red chromogen and hematoxylin counterstain.

### Histological scoring of pre-clinical anti-CD40 colitis model

Mouse colons were fixed in 10% neutral buffered formalin, dehydrated through graded alcohols, and embedded in paraffin blocks per standard protocols. 4 micron sections were collected on Superfrost plus glass slides. Following deparaffinization, tissues were stained with hematoxylin and eosin (H&E) dyes. A composite score was generated manual pathologist review, with a score assigned to each anatomical colon segment (proximal-, mid- and distal colon, and rectum), resulting in a summed score of 0–16 according to the following grading system: 0-Normal, 1-Increased lamina propria inflammation with or without associated crypt hyperplasia, but lacking crypt dropout. 2-Focal crypt dropout. 3-Moderate patchy crypt dropout. 4-Confluent crypt dropout with or without associated flattening of epithelial folds.

### Immunofluorescence

C57BL/6N mice aged 8 weeks were euthanized and the entire colon was dissected and placed in ice-cold PBS. Regions containing visible lymphoid aggregates were identified under a dissecting microscope and carefully excised. Tissue segments were fixed in 10% neutral buffered formalin for 2–4 hours at room temperature, followed by three washes in PBS (10 minutes each).Fixed tissues were opened along the mesenteric border and mounted flat with the mucosal surface facing up to visualize the epithelial layer of lymphoid aggregates en face. Whole mount preparations were incubated with Anti-GP2 antibody (Medical & Biological Laboratories, #D278-A48, dilution 1:1000) in PBS containing 1% BSA overnight at 4 °C in a humidified chamber. Following primary antibody incubation, tissues were washed three times in PBS (15 minutes each) and mounted using ProLong™ Gold Antifade Mountant (Invitrogen, #P10144) with coverslips. Images were acquired using a Nikon ECLIPSE Ti Series microscope and processed with Nikon imaging software.

### RNAscope

Colon tissues containing lymphoid aggregates from C57BL/6N mice were fixed in 10% neutral buffered formalin and embedded in paraffin. Sections (2 μm) were cut and mounted on Superfrost Plus slides. RNAScope 2.5 HD assay was performed according to the manufacturer’s protocol (Advanced Cell Diagnostics, #322350). Slides were baked at 60 °C for 60 minutes, then deparaffinized with xylene (5 minute, twice) followed by 100% ethanol (3 minute, twice) and 95% ethanol (3 minute, twice). Following antigen retrieval and protease treatment per manufacturer’s instructions, sections were hybridized with Probe-Mm-Gp2 (Advanced Cell Diagnostics, #474641) and developed using the RNAScope 2.5 HD Detection Reagent-RED. Sections were counterstained with hematoxylin (Abcam, #ab245880) and mounted with EcoMount. Images were acquired using a Nikon ECLIPSE Ti Series microscope under bright field settings and processed with Nikon imaging software.

### Isolation of the cells from colon

For colon digestion, colon tissues were first washed with Phosphate-Buffered Saline (PBS) to remove mucus and fecal residue. The tissues were then subjected to a non-enzymatic digestion with 10 mL of RPMI supplemented with 1% fetal bovine serum FBS, 20 mM EDTA and 1 mM Dithiothreitol (DTT). After incubating on a shaker at 37 °C for 30 minutes, the epithelial cell layer was removed by vigorous shaking for 15 seconds. The colon pieces were then resuspended in 10 mL RPMI medium supplemented with 1% FBS, 2 mg/ml collagenase II (Gibco, #17101015), 0.5 mg/ml Dispase (Gibco, #17105041) and 100 µg/ml Dnase I (Roche, #10104159001). The mixture was incubated at 37 °C while shaking for an hour. The resulting cell suspension was filtered through a 70 µm cell strainer for downstream flow cytometry analysis. When intracellular staining of cytokines is required, cells were stimulated *in vitro* with 50ng/ml phorbol 12-myristate 13-acetate (PMA) (Sigma, #P1585) and 1ug/ml ionomycin (Sigma, #I0634) in the presence of Golgiplug (BD Bioscience, #555029) for 3hr in a cell culture incubator (37 °C, 5% CO_2_).

### Flow cytometry

Cells isolated from colon tissues were resuspended in a 96-well round-bottom plate and incubated with FACS buffer containing Fc block (1:10 with Miltenyi, #130-092-575) for 20 minutes. After blocking, primary antibodies at 1:400 dilution and Live/Dead staining (Invitrogen, #L34955) at 1:2000 dilution was added and incubated for 30 minutes. Cells were then washed three times with FACS buffer, centrifuging at 300 x g for 5 minutes after each wash. For intracellular staining, Foxp3 staining kit (Invitrogen, #00-5523-00) was employed and procedures were followed according to the manufacturer’s manual. Flow cytometry data were acquired using a BD FACSymphony A5 Cell Analyzer with FACSDiva software and analyzed with FlowJo software (Tree Star). A detailed gating strategy is included in **Supplementary Figure 5**. Neutrophils are defined as alive CD45^+^, CD11b^+^, Siglec-F^-^, Ly6G^+^. Macrophages are defined as alive CD45^+^, CD11b^+^, Ly6G^-^, Siglec-F^-^, CD64^+^. CD4 T cells are defined as alive CD45^+^, CD4^+^, CD8a^-^, CD19^-^, TCRβ^+^. CD19 B cells are defined as alive CD45^+^, TCRβ^-^, CD19^+^. ILCs are defined as alive CD45^+^, CD90^+^, Lineage (CD3e, CD4, CD8, CD11b, CD11c, CD19, TCRβ, TCRγ/δ, NK1.1, GR1, Siglec-F, FcϵRI, Ter119) negative. Antibodies used in this study include: anti-CD45 (Biolegend, #103116), anti-CD11b (Thermo, #363-0112–82 or Biolegend, #101208), anti-Siglec-F (Biolegend, #155506), anti-Ly6G (Biolegend, #127608 or #127618), anti-CD11c (Biolegend, #117308), anti-CD4 (Biolegend, #100408 or Thermo, #46-0041-82), anti-CD8 (Biolegend, #100744 or #100708), anti-CD19 (Biolegend, #152408), anti-TCRβ (BD, #742785 or Biolgend, #109208), anti-CD90 (Biolegend, #140304), anti-TCRγ/δ (Biolegend, #118108), anti-NK1.1 (Biolegend, #156504), anti-FcϵRI (Biolegend, #134308), anti-Ter119 (Biolegend, #116208), anti-CD3e (BD, #565992), anti-GR1 (Biolegend, #108408), anti-B220 (Biolegend, #103208 or BD, #612838), anti-IL-17A (Biolegend, #506904), anti-IFNγ (BD, #612769), anti-RORγT (Thermo, #12-6981-82), anti-T-bet (Thermo, #53-5825-82), anti-Foxp3 (Thermo, #17-5773-82).

### Tissue homogenate for Luminex

For cytokine measurements by Luminex platform, cecal or colonic tissues were collected and incubated on wet ice for 5 minutes in 1x Cell Lysis Buffer (Cell Signaling, #9803) containing cOmplete protease inhibitor cocktail (Roche, #11697498001), at a concentration of 100mg tissue per 1ml lysis buffer. The samples were homogenized using a TissueLyser III (QIAGEN) at 30 Hz for 3 minutes. Lysates were centrifuged twice at 15,000 × g for 10 minutes at 4 °C to remove debris. The processed supernatant samples were diluted at 1:8 and analyzed for cytokine and chemokine levels using the MILLIPLEX^®^ Mouse Cytokine/Chemokine Panel (Thermo).

### RNA isolation and qPCR

Colon tissues containing lymphoid aggregates from C57BL/6N mice were collected and immediately placed in RLT buffer from the RNeasy Midi Kit (Qiagen, #75144). Tissues were homogenized by passing through a needle and syringe at least 30 times. Total RNA was extracted using the RNeasy Midi Kit according to the manufacturer’s protocol. RNA concentration and purity were determined using a NanoDrop spectrophotometer.

qPCR was performed using the TaqMan™ RNA-to-CT™ 1-Step Kit (Applied Biosystems, #4392938) on a QuantStudio 6 Real-Time PCR System (Applied Biosystems). TaqMan probes used were: GP2 (Mm00482557_m1), CCL20 (Mm01268754_m1), Sox8 (Mm00803422_m1), and β-actin (Mm01205647_g1) as housekeeping gene (Thermo Fisher Scientific). Gene expression was normalized to β-actin and analyzed using the ΔΔCt method.

### Single cell RNAseq analysis

Ileal and colonic epithelium UMAP embeddings and cell type labels from Thomas et al., 2024 were obtained from Zenodo (https://zenodo.org/records/14007626). The t-SNE embedding and cell type labels from Smillie et al., 2019 (32) were obtained from the Broad Single Cell Portal (https://singlecell.broadinstitute.org/single_cell/study/SCP259). The M cell signature score was calculated using AUCell (49) using the genes CCL20, CCL23, AIF1, TNFRSF11A, GP2, FERMT1, SPIB, SOX8, PTGER4, SH2B3, AHR. Expression dot plots were created using scater (50). Differential expression analysis in relative panels was performed using EdgeR (51).

### Statistical analysis

All statistical analyses were performed using GraphPad Prism or built-in R functions. For comparisons between two groups, unless otherwise specified, Mann-Whitney *U* tests were used to assess statistical significance. For comparisons involving more than two groups, a nonparametric one-way ANOVA (Kruskal-Wallis test) with Dunn’s multiple comparisons test was used as appropriate. For body-weight related analysis, RM two-way ANOVA with Sidak’s multiple comparison tests were performed. Statistical significance thresholds were set at P < 0.05, and all data are reported as mean ± SD (except for body weight data which are reported as mean ± SEM), and denoted as follows: ns, not significant; *,P < 0.05; **,P < 0.01; ***,P < 0.001; ****,P < 0.0001.

## Results

### Intestinal epithelial noncanonical NF-κB pathway is required for M-cell-mediated regulation of ILC3 and CD4^+^ T cells

**Figure 1:**
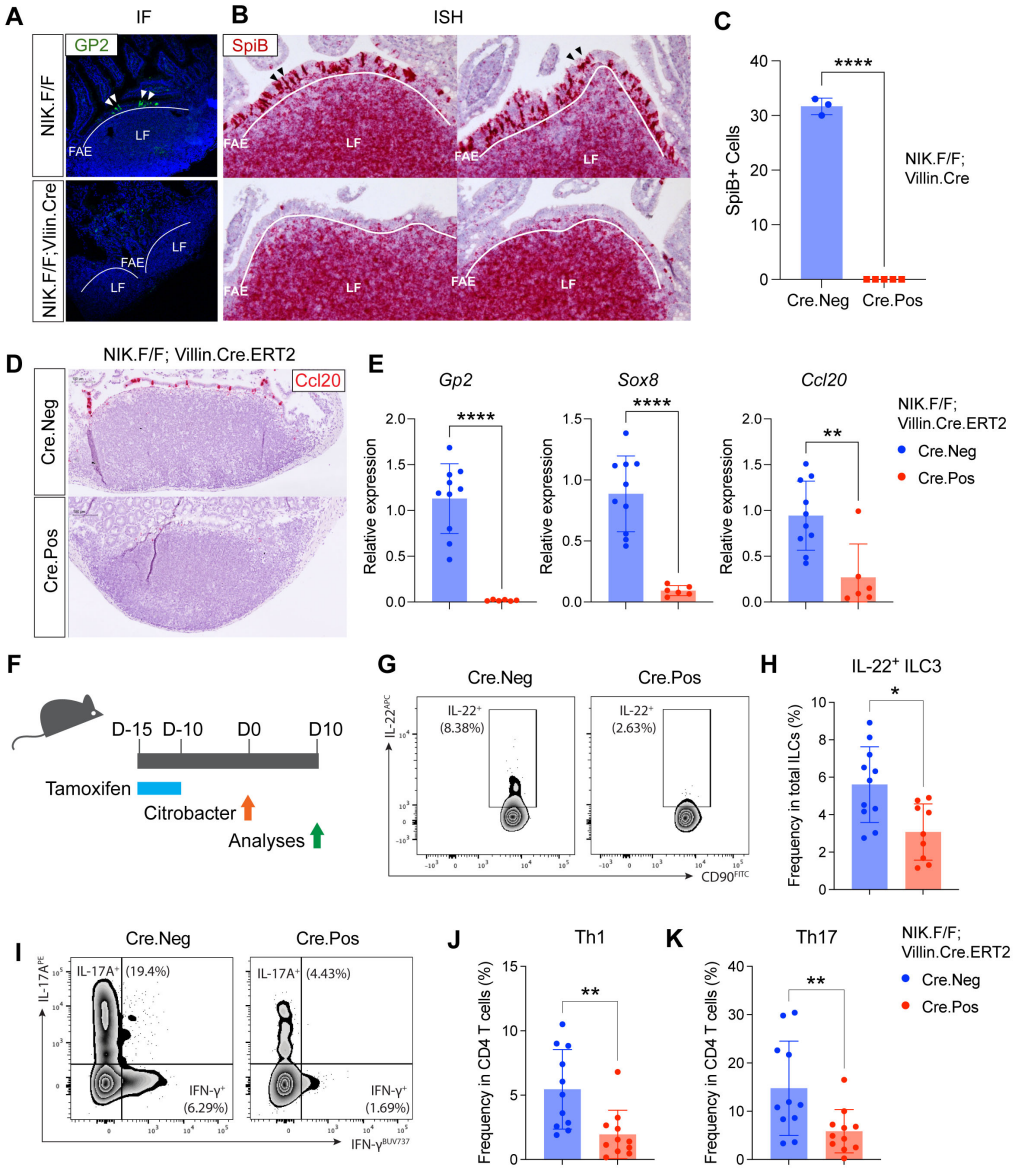
Intestinal epithelial noncanonical NF-κB pathway is required for M-cell-mediated regulation of ILC3 and CD4^+^ T cells. **(A-C)** Immunofluorescence staining of GP2 protein **(A)**, RNAscope *in situ* hybridization (ISH) of *SpiB* **(B)**, and quantification of the number of *SpiB*-expressing cells **(C)** in the Peyer’s patches of NIK.F/F and NIK.F/F;Villin.Cre mice. FAE: follicle-associated epithelium; LF: lymphoid follicle; arrowheads indicate M-cells. **(D, E)** RNAscope ISH of *Ccl20* **(D)** and quantitative PCR analysis of *Gp2*, *Sox8*, and *Ccl20* expression **(E)** in the Peyer’s patches of NIK.F/F and NIK.F/F;Villin.Cre.ERT2 mice. **(F-K)** NIK.F/F and NIK.F/F;Villin.Cre.ERT2 mice were compared in a *Citrobacter rodentium* infection model. Experimental schematic of the infection setup **(F)**. Flow cytometric analysis **(G)** and quantification **(H)** of IL-22-expressing ILC3-like cells (defined as CD45^+^CD90^+^Lineage^-^; gating strategy in **Supplementary Figure 5C**). Flow cytometric analysis **(I)** and quantification of Th1 **(J)** and Th17 cells **(K)** in the colon lamina propria. Data are based on analyses of 3 to 11 mice per group. Statistical significance is presented as mean ± SD. Unpaired t-tests with Welch’s correction **(C, E)** or Mann-Whitney *U* tests **(H, J, K)** were performed. Statistical significance is indicated as follows: *, P < 0.05; **, P < 0.01; ***, P < 0.001; ****, P < 0.0001.

The noncanonical NF-κB pathway in IECs is essential for the differentiation and maintenance of microfold cells (M-cells), a subgroup of specialized follicle associated epithelium (FAE) cells critical for gut immune surveillance. M-cell differentiation is driven by the RANK-RANKL axis, which activates NIK signaling (21, 29) in IECs. To investigate the role of this pathway, we utilized NIK conditional knockout mice with gut epithelial cell-specific deletion driven by Villin-cre (NIK.F/F; Villin.Cre). These mice exhibited a complete absence of M-cells in the Peyer’s patches, as demonstrated by immunofluorescence staining for the M-cell surface antigen GP2 (30) (**Figure 1A**) and *in situ* hybridization for the transcription factor SpiB (31) (**Figures 1B, C**).

To assess the role of NIK in adult mice, we employed a tamoxifen-inducible Villin-Cre.ERT2 system (NIK.F/F; Villin.Cre.ERT2) to delete NIK in IECs. Inducible knockout of NIK in adult mice led to the loss of M-cells, as indicated by the downregulation of signature M-cell marker genes such as *Gp2*, *Ccl20*, and *Sox8* in Peyer’s patches (**Figures 1D, E**). Moreover, we identified that M-cells are not restricted to the small intestinal Peyer’s patches but are also present in colonic lymphoid patches (**Supplementary Figure 1A**). Treatment with anti-RANKL antibody resulted in the depletion of M-cells, confirming that RANK-NIK signaling axis is essential not only for the differentiation of M-cells but also for their maintenance in adult mice (**Supplementary Figures 1B, C**).

M-cells are specialized for sampling luminal microbiota and facilitating trans-epithelial transport, providing a direct contact between luminal antigen and immune cells residing in the lymphoid follicles (32). To evaluate the functional implications of M-cell depletion on gut immune responses, we utilized a model of *Citrobacter rodentium*-induced colitis (**Figure 1F**). In the absence of M-cells, we observed a significant reduction of IL22-expressing ILC3-like cells (CD45^+^CD90^+^Lineage^-^) in the lamina propria (**Figures 1G, H**). Similarly, Th1 and Th17 populations were also markedly reduced (**Figures 1I–K**). Interestingly, despite these cellular changes, the depletion of M-cells did not lead to overt alterations in body weight, colon length, or colon density (colon weight-to-length ratio, reported as mg/cm, where higher values reflect edema, immune cell infiltration, and tissue thickening), as measured by the weight-to-length ratio (**Supplementary Figure 1D**). We note that fecal or tissue *Citrobacter rodentium* CFU were not measured in this experiment; therefore, we cannot exclude the possibility that differences in pathogen burden contributed to the altered ILC3 and CD4^+^ T cell responses observed in M-cell–deficient mice.

The observed reduction in the innate and adaptive lymphocyte populations is likely attributable to the loss of M-cell-produced CCL20, a key chemokine that recruits CCR6-expressing cells, including ILC3 and Th17. Together, these findings underscore the critical role of the IEC-intrinsic noncanonical NF-κB pathway in M-cell differentiation and maintenance, highlighting its importance in regulating lymphocyte responses during intestinal inflammation.

### M-cells are present in the human small and large intestines and dysregulated in IBD

**Figure 2:**
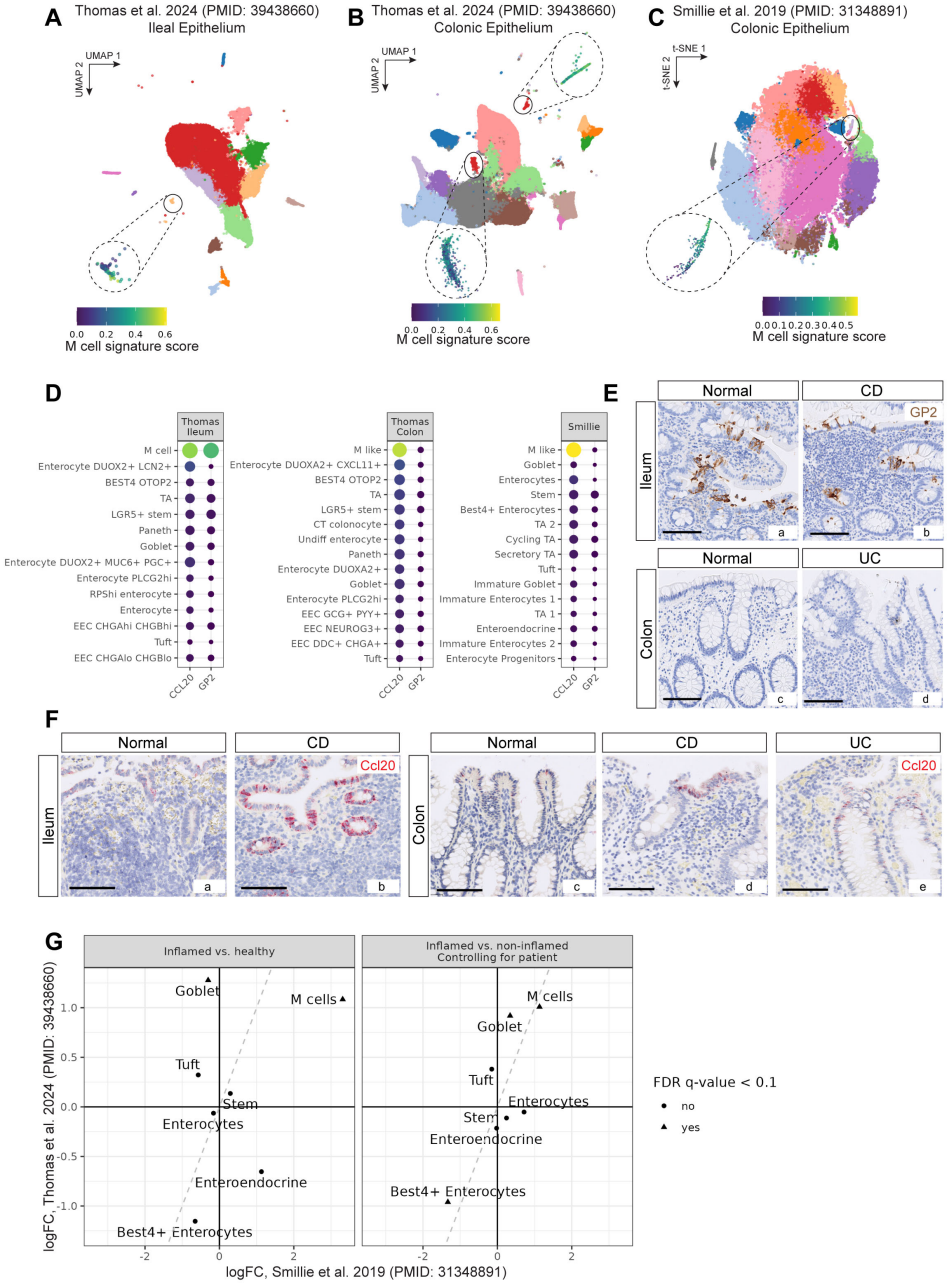
M-cells are present in the human small and large intestine and are dysregulated in IBD. **(A, B)** UMAP plot of the ileal **(A)** and colonic epithelium **(B)** from Thomas et al., 2024 (PMID: 39438660), highlighting a population of cells with high M-cell signature scores. The dashed circle zooms in on the cells outlined by the solid line. **(C)** t-SNE plot of colonic epithelium from Smillie et al., 2019 (PMID: 31348891), highlighting cells annotated as M cells. **(D)** Expression of *CCL20* and *GP2* in M-cell populations identified within the Thomas ileal epithelium dataset, using M-cell signature scores, as well as in author-annotated cell types from the respective datasets. **(E)** Anti-GP2 immunohistochemistry on human intestinal biopsies. Panels (a, b) ileum. (a) Normal tissue; (b) biopsy from a Crohn’s disease patient. Panels (c, d) colon. (c) Normal tissue; (d) biopsy from a patient with ulcerative colitis. Scale bar = 100 µm. (F) *CCL20 in situ* hybridization on human intestinal resections. Panels a-b: ileum. (a) Normal tissue; (b) tissue from a Crohn’s disease patient. Panels (c-e) colon. (c) Normal tissue; (d) tissue from a Crohn’s disease patient; (e) tissue from a patient with ulcerative colitis. Scale bar = 100 µm. **(G)** Differential abundance analysis of colonic epithelial cell types. Left panel: log-fold changes in abundance between inflamed and healthy samples. Right panel: log-fold changes in inflamed versus non-inflamed samples. The dashed line corresponds to y=x. Triangles indicate cell types passing FDR q-value < 0.1.

The dysregulation of M-cells has significant implications in the context of IBD. While M-cells are generally rare in healthy colon tissue, their proportion increases during inflammation, particularly in UC, where they potentially contribute to disease progression (33, 34). To further investigate the role of M-cells in IBD, we re-analyzed two publicly available single-cell RNA sequencing (scRNA-seq) datasets that included epithelial cells from both the ileum and colon (33, 35). Using a curated gene signature to identify M-cell populations within these datasets, we confirmed the presence of M-cells in both the ileum and colon (**Figures 2A-C** and **Supplementary Figures 2A–C**), consistent with findings in the murine models (**Figure 1A** and **Supplementary Figure 1A**).

Interestingly, we observed a key distinction between ileal and colonic M-cells based on their marker expression. Ileal M-cells abundantly express GP2, a canonical marker of M-cells, at both the RNA level and protein levels. In contrast, colonic M-cells lack GP2 expression (**Figures 2D, E**). Despite this divergence in GP2 expression, both ileal and colonic M-cells express the chemokine CCL20 (**Figures 2D, F**), a feature that underscores their conserved functional role in recruiting CCR6-expressing immune cells, such as ILC3 and Th17 cells. Importantly, we also observed that M-cells in both the ileum and colon are closely associated with the lymphoid aggregates (**Figures 2E, F**).

In the context of IBD, our analysis revealed that CCL20-expressing M-cells are elevated in both the ileum and colon of IBD patients compared to healthy controls (**Figure 2F**). Furthermore, scRNA-seq data consistently demonstrated that M-cells populations are significantly increased in IBD patients compared to healthy control, as well as in inflamed regions compared to non-inflamed regions from the same patients across two independent datasets (**Figure 2G**). These findings suggest that M-cell dysregulation, characterized by an increase in their proportion and chemokine expression, is a hallmark of the inflamed active disease in IBD.

### Dendritic cell-specific noncanonical NF-κB pathway contributes to gut inflammation

**Figure 3:**
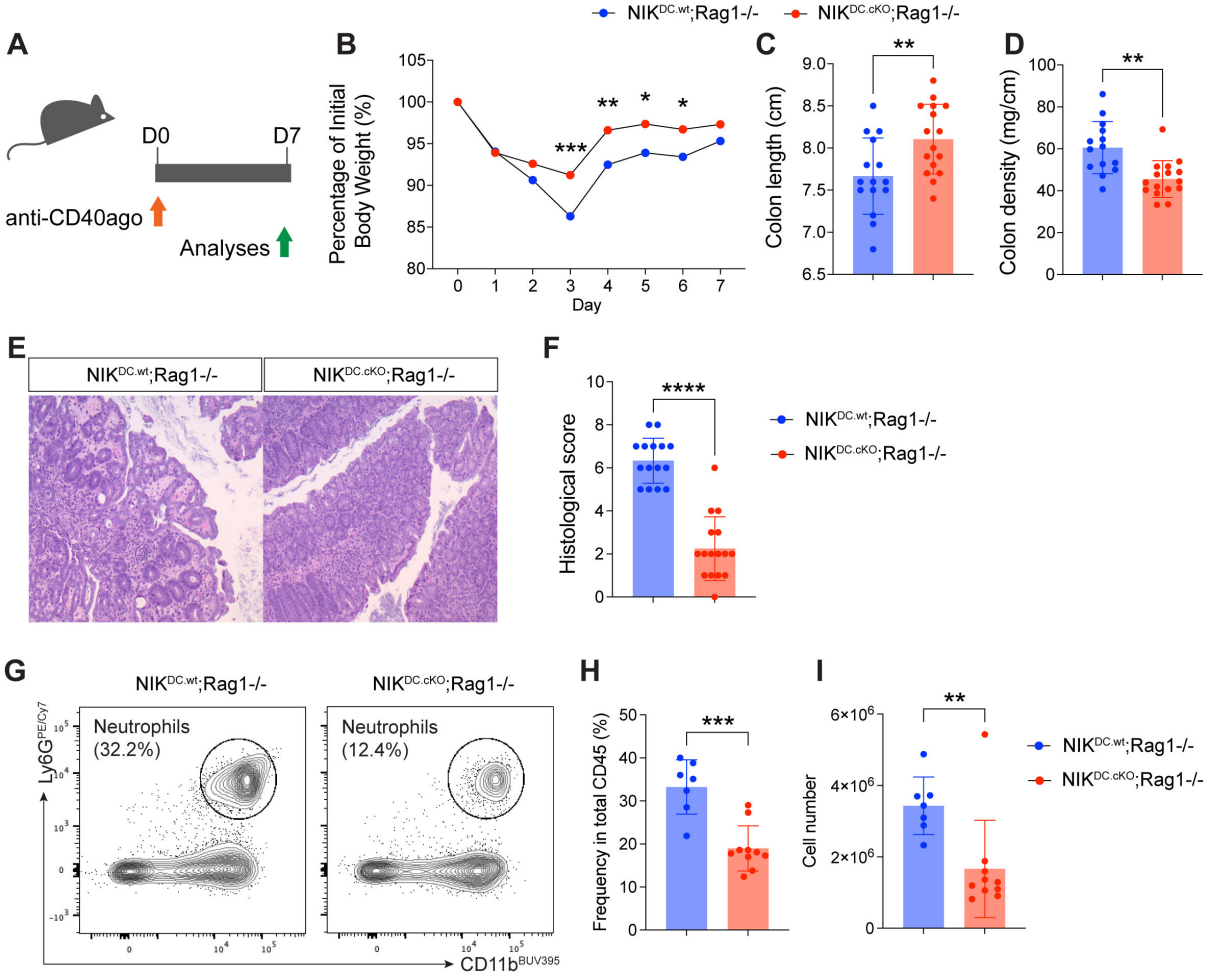
Dendritic cell-specific noncanonical NF-κB pathway contributes to gut inflammation. **(A)** Experimental schematic for the anti-CD40 agonist-induced colitis model comparing NIK.F/F;Rag1-/- and NIK.F/F;CD11c.Cre;Rag1-/- mice. **(B-D)** Assessment of colitis severity in the two groups, including body weight changes **(B)**, colon length **(C)**, and colon density (colon weight-to-length ratio) **(D)**. **(E, F)** Representative histological images of colon tissue **(E)** and histological scoring of inflammation severity **(F)** in NIK.F/F;Rag1-/- and NIK.F/F;CD11c.Cre;Rag1-/- mice. **(G-I)** Flow cytometric analysis of neutrophil infiltration in the colon lamina propria. Representative flow cytometry plots **(G)**, quantification of neutrophil frequency **(H)**, and total neutrophil numbers **(I)** are shown (neutrophils defined as alive CD45^+^CD11b^+^Ly6G^+^; gating strategy in **Supplementary Figure 5A**). Data are based on analyses of 7 to 15 mice per group. Statistical significance is presented as mean ± SD. RM two-way ANOVA (B) or Mann-Whitney *U* tests were applied where appropriate. Statistical significance is indicated as follows: *, P < 0.05; **, P < 0.01; ***, P < 0.001; ****, P < 0.0001.

The noncanonical NF-κB pathway plays a pivotal role in dendritic cells (DC), where it amplifies immune responses by regulating cytokine and chemokine production. For example, the NIK-activating receptor CD40 is well-known for its ability to trigger robust inflammatory signaling in dendritic cells (17). CD40 and its ligand, CD40L, have been widely implicated in the pathogenesis of IBD, with elevated expression observed in intestinal lesions of IBD patients (36, 37). To investigate the specific role of the noncanonical NF-κB pathway in dendritic cells during gut inflammation, we utilized the anti-CD40 agonistic antibody (anti-CD40ago)-induced colitis model (**Figure 3A**). We employed NIK conditional knockout mice with CD11c-Cre on a Rag1-deficient background (NIK^DC.cKO^; Rag1-/-), thus bypassing the contribution of B cells, which also express CD40, and focusing on CD11c-expressing DCs.

Compared to the Cre-only control mice (NIK^DC.wt^; Rag1-/-), NIK^DC.cKO^; Rag1-/- mice exhibited significantly attenuated colitis following anti-CD40ago treatment. This was demonstrated by reduced body weight loss (**Figure 3B**), improved colon length, and decreased colon density (colon weight-to-length ratio) (**Figures 3C, D**). Histological analysis further revealed minimal immune cell infiltration into the colonic lamina propria of NIK^DC.cKO^; Rag1-/- mice (**Figure 3E**) and significantly lower histological scores compared to control mice (**Figure 3F**). Neutrophils, a hallmark of active gut inflammation, were profiled in the colon lamina propria (LP) through flow cytometry at day 7 post anti-CD40ago treatment. In NIK^DC.cKO^; Rag1-/- mice, the number of neutrophils (Ly6G+CD11b+) was markedly reduced compared to control mice (**Figures 3G–I**). These results indicate that NIK activity in dendritic cells is essential for driving colitis in this model.

### CD4^+^ T cell-specific noncanonical NF-κB pathway contributes to gut inflammation

The noncanonical NF-κB pathway within CD4^+^ T cells plays a critical role in gut inflammation, with the co-stimulatory receptor OX40 being a key activator of this pathway. OX40 signaling is widely implicated in the pathogenesis of IBD (4, 38) and is essential for T cell activation, proliferation, and survival (39). Importantly, OX40 activity relies almost entirely on NIK signaling (40). To specifically interrogate the role of NIK in CD4^+^ T cells during gut inflammation, we employed the adoptive T cell transfer colitis model.

**Figure 4:**
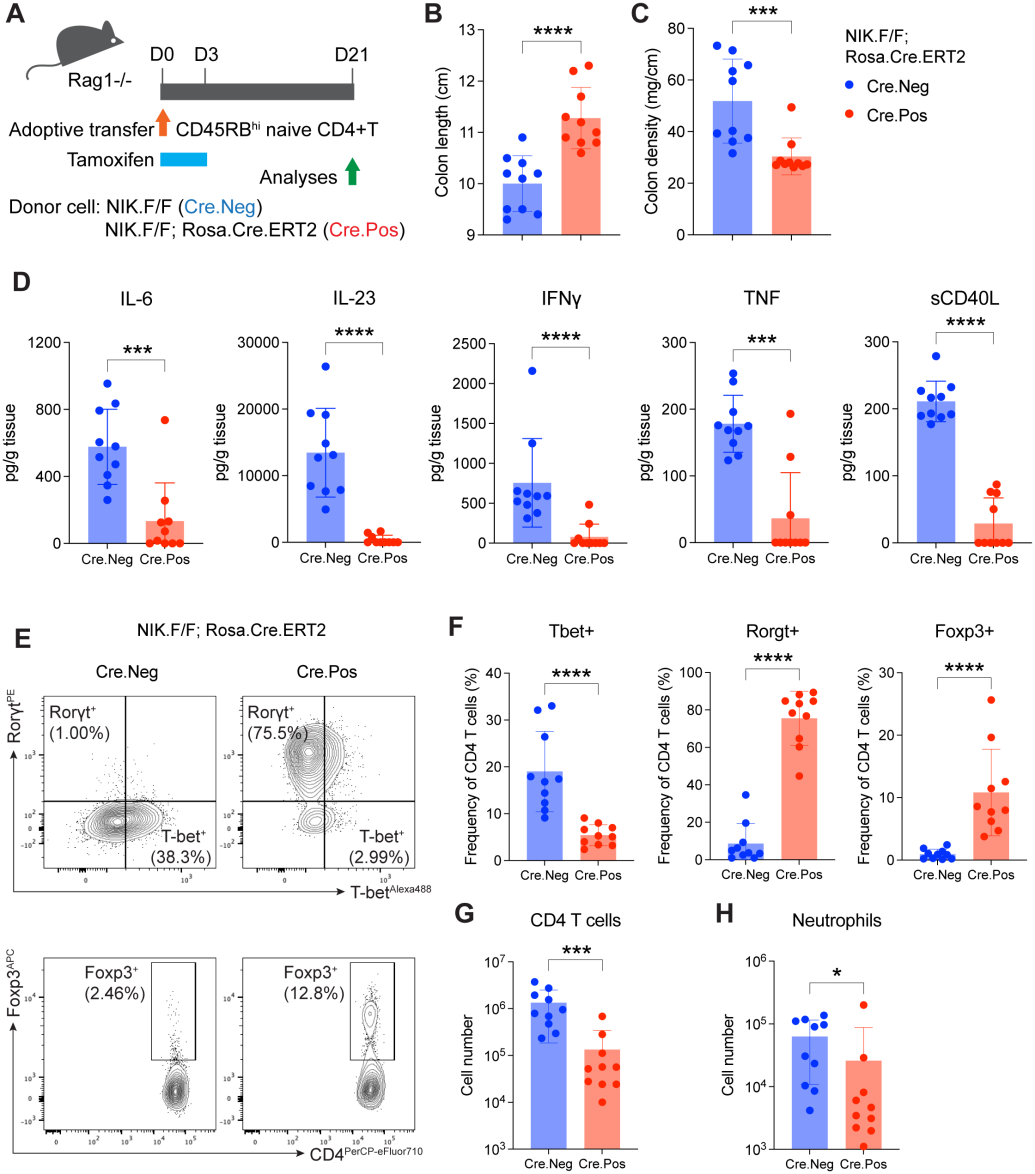
T cell-specific noncanonical NF-κB pathway contributes to gut inflammation. **(A)** Experimental schematic for the adoptive T cell transfer-induced colitis model. CD45RB^hi^ naïve CD4+ T cells from NIK.F/F or NIK.cKO;Rosa.Cre.ERT2 donor mice were transferred into Rag1-/- recipient mice, with tamoxifen treatment administered to induce NIK deletion in donor T cells. **(B, C)** Assessment of colitis severity in recipient mice, including colon length **(B)** and colon density (colon weight-to-length ratio) **(C)**. **(D)** Cytokine profiles of colon tissues, including TNF, IL-23, IL-6, IFNγ, and soluble CD40L (sCD40L). **(E, F)** Flow cytometric analysis of T-bet+ (Th1), RORγt+ (Th17), and Foxp3+ (Treg) CD4+ T cell populations **(E)**, with quantification of the relative frequencies of these cells in the CD4 compartment **(F)**. Gating strategy is shown in **Supplementary Figure 5B**. **(G, H)** Quantification of total cell numbers of CD4+ T cells **(G)** and neutrophils **(H)** in the colon lamina propria. Data are based on analyses of 10 mice per group. Statistical significance is presented as mean ± SD. Mann-Whitney *U* tests were applied where appropriate. Statistical significance is indicated as follows: *, P < 0.05; **, P < 0.01; ***, P < 0.001; ****, P < 0.0001.

In this model, CD45RB^hi^ naïve CD4^+^ T cells were isolated from either control Cre.Neg (NIK.F/F) mice or Cre.Pos mice (NIK.F/F; Rosa.Cre.ERT2). These donor T cells were transferred into Rag1-deficient recipient mice to induce colitis. To ablate NIK in the donor T cells, tamoxifen treatment was administered following the transfer (**Figure 4A**). Strikingly, mice that received Cre.Pos NIK-deficient T cells developed significantly less severe colitis compared to those that received control Cre.Neg T cells. This was evidenced by improved colon length and reduced colon density in the recipient mice (**Figures 4B, C**). Additionally, the levels of pro-inflammatory cytokines, including TNF, IL-23, IL-6, IFNγ, and soluble CD40L, were markedly reduced in mice receiving NIK-deficient T cells (**Figure 4D**).

At the cellular level, we observed a profound shift in T cell differentiation. Donor T cells lacking NIK were more likely to differentiate into Foxp3-expressing regulatory T cells (Tregs). Within effector T cell populations, control T cells were predominantly skewed toward T-bet-expressing Th1 cells, while NIK-deficient T cells differentiated into Rorgt-expressing Th17 cells, which were reported to play protective roles in adoptive transfer colitis model (41) (**Figures 4E, F**). Furthermore, the total number of CD4^+^ T cells in the lamina propria was significantly reduced in mice receiving NIK-deficient T cells (**Figure 4G**), accompanied by a parallel reduction in neutrophil infiltration (**Figure 4H**). These findings demonstrated that NIK activity in CD4^+^ T cells promotes the differentiation of inflammatory Th1 cells over Th17 or the immune suppressive Tregs cells, thus amplifying the inflammatory response and exacerbating colitis.

### Global NIK deletion reduces gut inflammation in a complex colitis model

**Figure 5:**
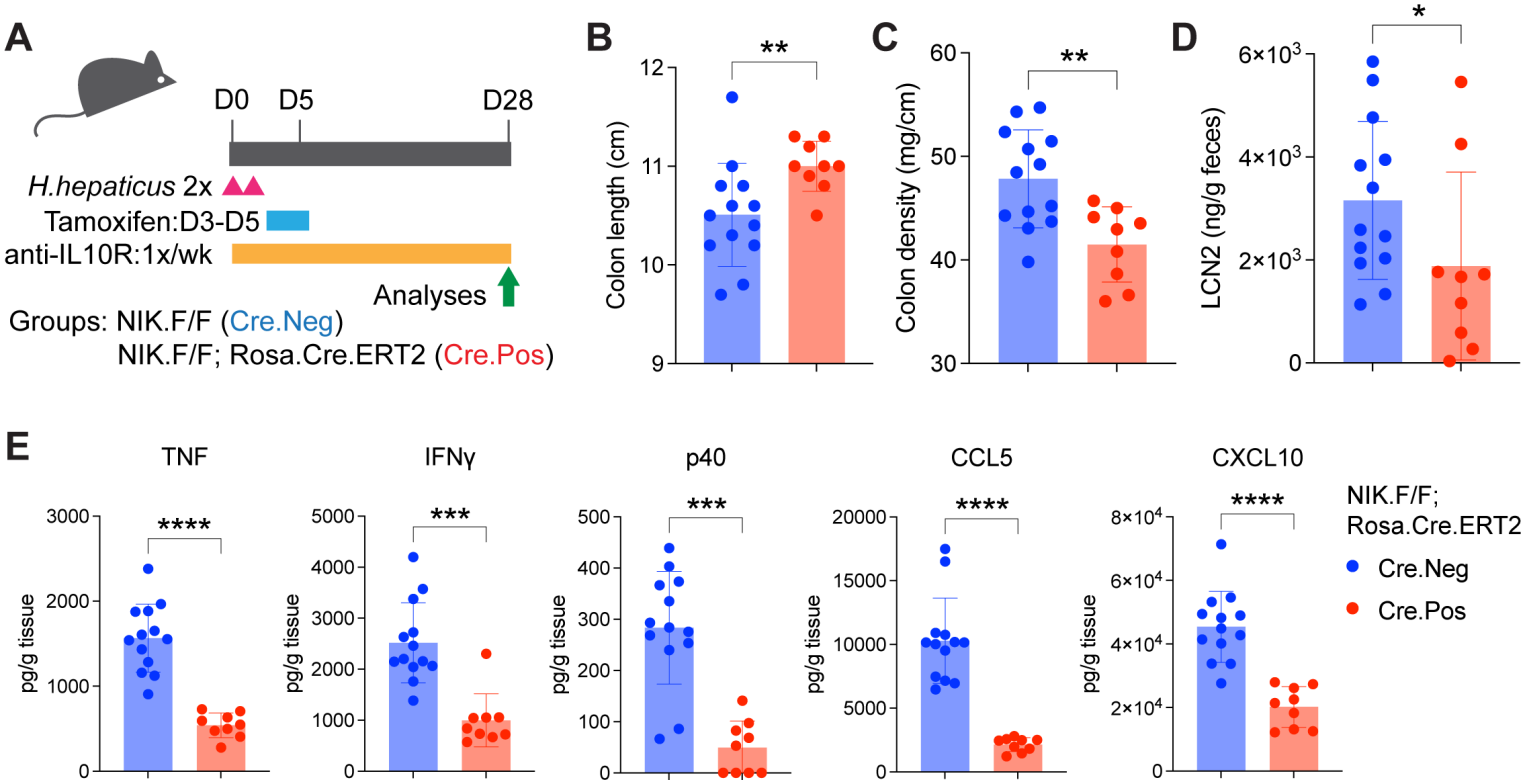
Global NIK deletion reduces gut inflammation in a complex colitis model. **(A)** Experimental schematic for the *Helicobacter hepaticus* + anti-IL-10R colitis model. *H. hepaticus* was administered by oral gavage on days 0 and 1 to NIK.F/F and NIK.cKO;Rosa.Cre.ERT2 mice. Tamoxifen was administered daily from days 3 to 5 to induce systemic NIK deletion, and anti-IL-10R antibodies were injected weekly. Mice were analyzed on day 28. **(B-D)** Assessment of colitis severity in recipient mice. Colon length **(B)** and colon density (colon weight-to-length ratio) **(C)** were measured at the time of takedown. Fecal lipocalin-2 (LCN2) levels, an inflammatory marker, were assessed at week 2 **(D)**. **(E)** Cytokine profiles of colon tissues, including TNF, IFNγ, p40 (IL-12/23 subunit), CCL5, and CXCL10. Data are based on analyses of 9 to 13 mice per group. Statistical significance is presented as mean ± SD. Mann-Whitney *U* tests were applied where appropriate. Statistical significance is indicated as follows: *, P < 0.05; **, P < 0.01; ***, P < 0.001; ****, P < 0.0001.

Having established the critical role of the noncanonical NF-κB pathway within specific cellular compartments, we next sought to evaluate the whole-body impact of NIK deletion on gut inflammation. To this end, we again utilized the tamoxifen-inducible Rosa.Cre.ERT2 model to achieve systemic NIK deletion. Mice were tested in the *Helicobacter hepaticus* plus anti-IL10R colitis model, which represents a complex and multifactorial disease system (42). This model mimics key features of IBD, including microbial dysbiosis, autoantibody-mediated loss of immune tolerance, and chronic inflammation (**Figure 5A**).

In Cre.Pos (NIK.F/F; Rosa.Cre.ERT2) mice, we observed significantly attenuated gut inflammation compared to control Cre.Neg (NIK.F/F) mice in both the cecum and colon (**Figures 5B, C**). Stool samples revealed a reduction in lipocalin-2 (LCN2), a well-established marker of gut inflammation and epithelial damage, as early as week 2 (**Figure 5D**). Analysis of several inflammatory markers further supported the protective effects of global NIK deletion. Pro-inflammatory cytokines and chemokines, including TNF, IFNγ, IL-12b (p40), CCL5, and CXCL10, were significantly reduced in the colon tissues of NIK-deficient mice compared to controls (**Figure 5E**). Collectively, these findings demonstrate that systemic NIK deletion significantly mitigated gut inflammation in a complex colitis model and provide strong rationale for targeting NIK as a therapeutic strategy for IBD.

### Pharmacokinetic and pharmacodynamic characterization of a NIK inhibitor

**Figure 6:**
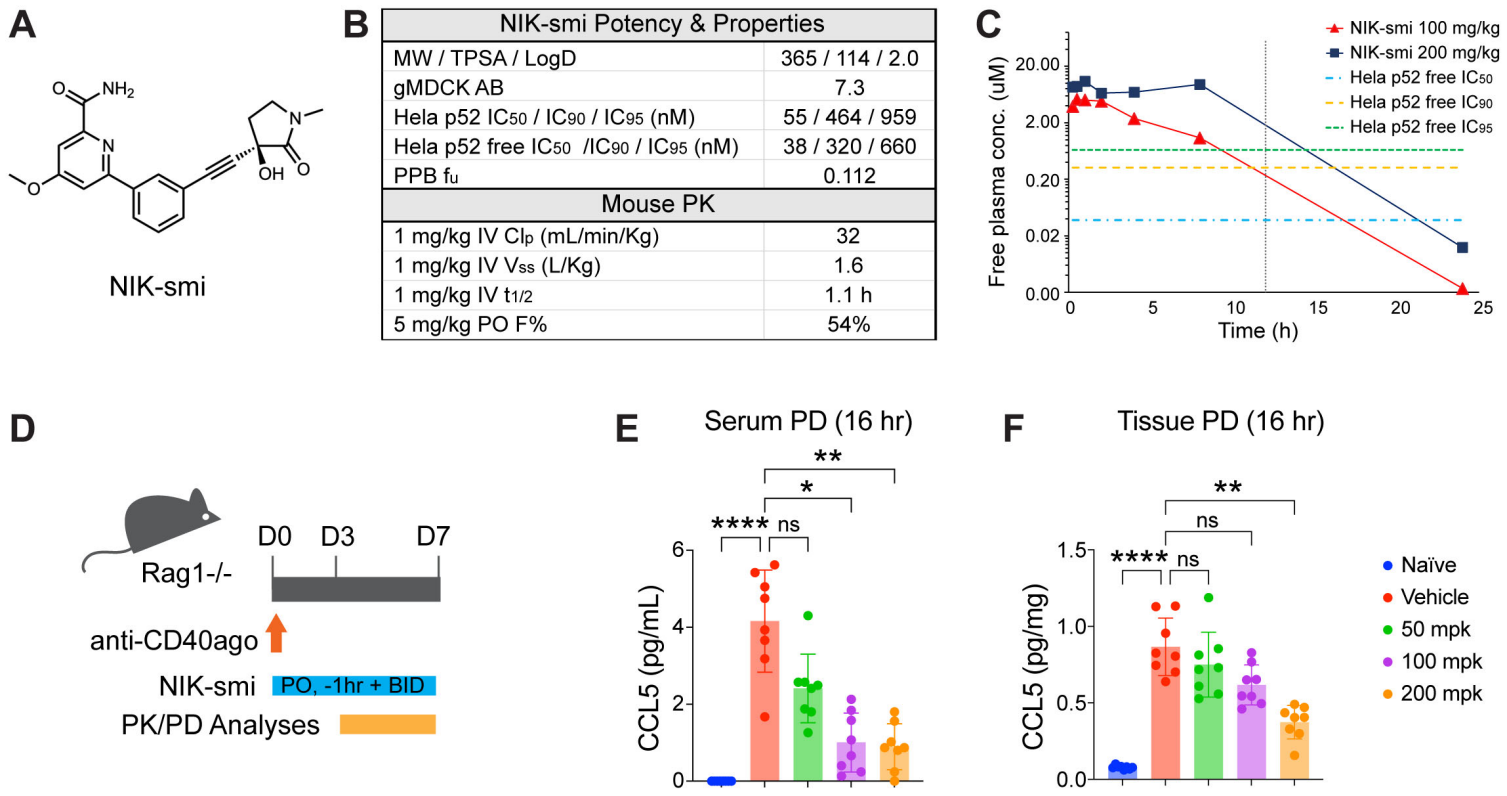
Pharmacokinetic and pharmacodynamic characterization of a NIK inhibitor. **(A)** Chemical structure of NIK-smi. **(B)** Summary of NIK-smi properties, including potency and PK parameters in mice. **(C)** PK profile of NIK-smi, showing free IC_50_, IC_90_, and IC_95_ coverage for single oral dose at 100 mg/kg and 200 mg/kg. **(D-F)** Experimental schematic of the anti-CD40 agonist (anti-CD40ago)-induced colitis model used to evaluate PD effects of NIK-smi **(D)**. Dose-dependent PD effects of NIK-smi, as measured by reductions in the chemokine CCL5, a biomarker of CD40-dependent activity, in serum **(E)** and tissue **(F)**. Data are based on analyses of 8 to 12 mice per group. Statistical significance is presented as mean ± SD. Kruskal-Wallis tests with Dunn’s multiple comparisons test **(E, F)**.Statistical significance is indicated as follows: *, P < 0.05; **, P < 0.01; ***, P < 0.001; ****, P < 0.0001.

To further explore the therapeutic potential of targeting NIK, we utilized a potent, selective small molecule inhibitor (NIK-smi) designed to specifically block NIK kinase activity (**Figure 6A**) (26). NIK-smi is an internally developed active-site inhibitor with excellent physiochemical properties and potency as shown in **Figure 6B** (43). Through our studies, we developed the HeLa-LTβR-induced p52 translocation assay (HeLa-p52) as the most representative measure of NIK-smi cellular activity. In this assay, NIK-smi exhibits double digit nM IC_50_ (**Figure 6B**). Based on our prior experience, we hypothesized that an efficacious dose would require 24-hour free unbound plasma coverage above the free IC_90_ of the HeLa-p52 assay. NIK-smi exhibits a free IC_90_ and IC_95_ potency of 320 nM and 660 nM, respectively. Low dose IV and PO administration of NIK-smi in mice demonstrates an acceptable pharmacokinetic (PK) profile of this compound, with a plasma clearance of 32 mL/min/kg, a volume of distribution of 1.6 L/kg, a half-life of 1.1 hours, and 54% oral bioavailability (**Figure 6B**). To evaluate whether this low dose PK would scale proportionally to higher doses required for pharmacodynamic (PD) and efficacy studies, we administered this compound to mice in single PO doses of 100 and 200 mg/kg. At 200 mg/kg, NIK-smi provided free HeLa-p52 IC_90_ and IC_95_ coverage for approximately 17 and 15 hours, respectively, while the 100 mg/kg dose achieved free IC_90_ for only 11 hours and IC_95_ for 9 hours (**Figure 6C**). Neither dose provided 24-hour coverage over IC_90_, indicating that a BID dosing regimen would be necessary for sustained NIK inhibition.

To establish a robust PK/PD model, we employed the anti-CD40ago antibody-induced colitis model (**Figure 6D**), leveraging the demonstrated dependency of this pathway on NIK (**Figure 3**). In this model, NIK-smi exhibited dose-dependent PD effects, evidenced by reduction in the chemokine CCL5, a key biomarker for CD40-dependent activity, in both serum and tissue (**Figures 6E, F**). PK/PD modeling further supported the requirement for a 200 mg/kg BID dosing regimen to achieve sustained NIK inhibition. This dosing strategy provided the foundation for subsequent efficacy studies in preclinical models.

### Pharmacologic inhibition of NIK demonstrated efficacy in multiple preclinical colitis models

**Figure 7:**
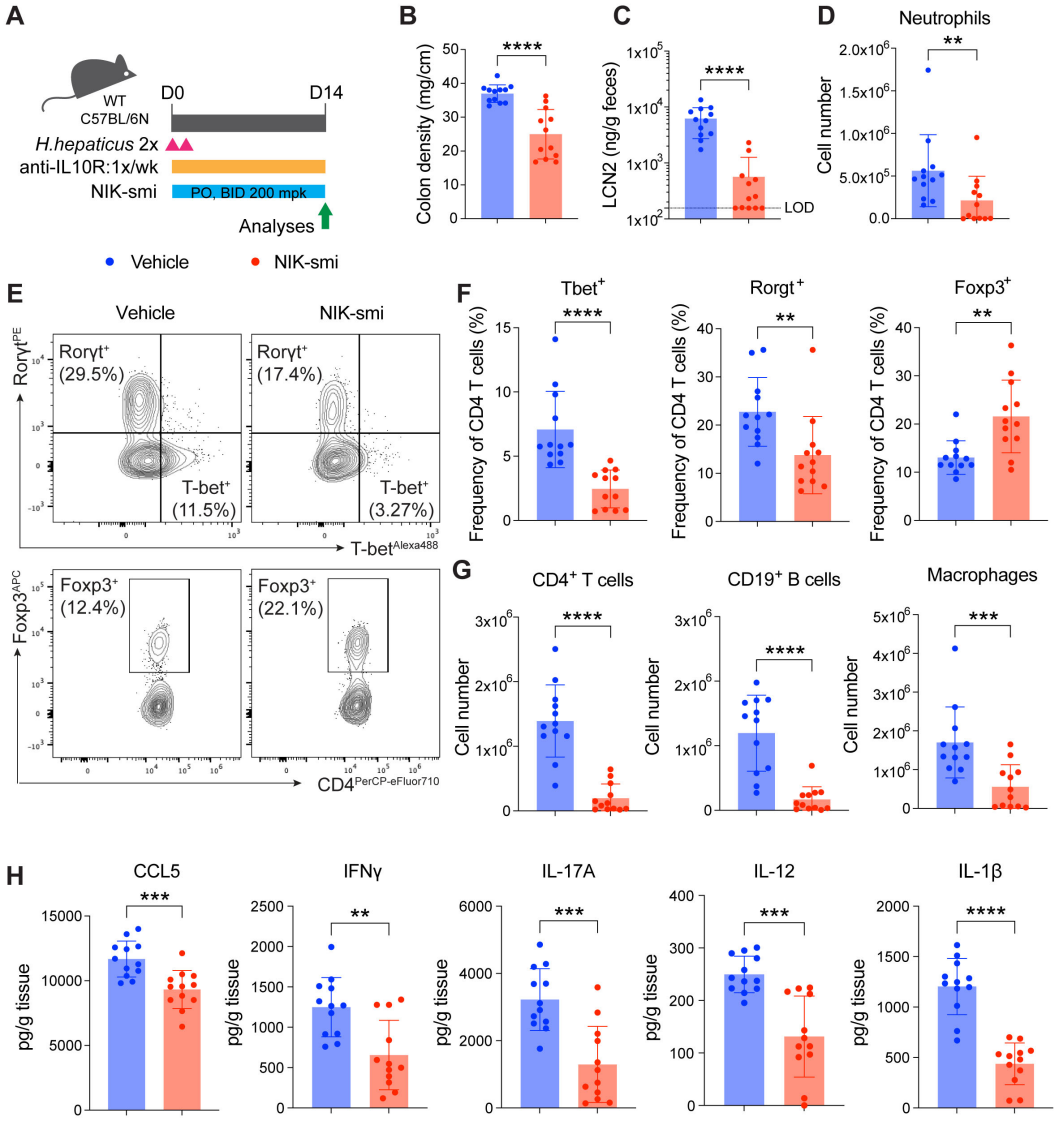
Pharmacologic inhibition of NIK demonstrated efficacy in a preclinical colitis model. **(A)** Experimental schematic for the *H. hepaticus* + anti-IL-10R colitis model. Wild-type C57BL6 mice were orally gavaged *H. hepaticus* on days 0 and 1, followed by weekly anti-IL10R antibody injections. Mice were treated with vehicle or NIK-smi (200 mg/kg BID via oral gavage) and analyzed on day 14. **(B, C)** Assessment of colitis severity. Colon density (colon weight-to-length ratio) was measured at the time of takedown **(B)**, and fecal lipocalin-2 (LCN2) levels were assessed at day 8 **(C)**. **(D)** Quantifications of neutrophil numbers in the gut lamina propria by flow cytometry. (gating strategies in **Supplementary Figure 5A**) **(E, F)** Flow cytometric analysis of RORγt+ (Th17) and T-bet+ (Th1) CD4+ T cell populations and Foxp3+ (Treg) cells **(E)**, along with quantification of their relative frequencies in the CD4+ T cell compartment **(F)**. Gating strategy is shown in **Supplementary Figure 5B**. **(G)** Quantification of total CD4+ T cell, CD19+ B cell, and macrophages in the gut lamina propria. (gating strategies in **Supplementary Figure 5B**) **(H)** Luminex analysis of cytokine profiles in colon tissues, including CCL5, IFNγ, IL-17A, IL-12, and IL-1β. Data are based on analyses of 12 mice per group. Statistical significance is presented as mean ± SD. Mann-Whitney *U* tests were applied where appropriate. Statistical significance is indicated as follows: *, P < 0.05; **, P < 0.01; ***, P < 0.001; ****, P < 0.0001.

Having established the PK/PD relationship of NIK-smi, we next evaluated its therapeutic efficacy in several preclinical models of colitis using the 200 mg/kg BID dosing regimen. We first tested NIK-smi in the *Helicobacter hepaticus* plus anti-IL10R colitis model (**Figure 7A**), a complex model involving diverse immune responses in immunocompetent mice (42). Consistent with the results observed in NIK global knockout mice (**Figures 5A–E**), treatment of the mice with NIK-smi significantly reduced intestinal inflammation. This was evidenced by reductions in colon density (**Figure 7B**), fecal LCN2 levels (**Figure 7C**) and infiltrating neutrophils (**Figure 7D**). Importantly, NIK-smi treatment skewed the CD4^+^ T cell phenotype, reducing effector Th1 while increasing Treg population (**Figures 7E, F**), a phenomenon also observed with NIK-deficient T cells (**Figures 4E, F**), additionally, total number of CD4^+^ T cells, B cells, and macrophages are reduced (**Figure 7G**). Furthermore, molecular analysis showed reduced levels of biomarker CCL5, as well as other cytokines such as IFNγ, IL-17A, IL-12, and IL-1β (**Figure 7H**), demonstrating the anti-inflammatory capacity in this model.

To further validate the efficacy of NIK-smi in other models, the adoptive T cell transfer colitis model was employed (**Supplementary Figure 3A**). Treatment of mice with NIK-smi significantly attenuated gut inflammation compared to vehicle-treated controls, as evidenced by improved colon length and reduced colon density (**Supplementary Figures 3B, C**). At the molecular level, NIK-smi suppressed pro-inflammatory cytokines such as TNF, IL-23, IFNγ, sCD40L, and the biomarker CCL5, reducing their levels to near-background in the colon tissues (**Supplementary Figure 3D**).

Both the *Helicobacter hepaticus* colitis model and the adoptive T cell transfer colitis model are characterized by IL-12/23-dependent Th1-driven immune responses, reflecting CD-like features. To further evaluate the therapeutic potential of NIK-smi in a model resembling UC, we tested it in the DSS-induced colitis model (**Supplementary Figure 4A**). NIK-smi treatment improved body weight recovery and enhanced colon health, as shown by increased colon length and decreased colon density (**Supplementary Figures 4B, C**). Flow cytometric analysis demonstrated reduced effector CD4^+^ T cell populations, including Th1 and Th17 cells, in the colonic tissue of NIK-smi-treated mice (**Supplementary Figure 4D**). Additionally, NIK-smi reduced levels of biomarker CCL5 and inflammatory cytokines such as IL-1β and IL-6 (**Supplementary Figure 4E**), further supporting its efficacy in this UC-like model.

Together, these findings demonstrate that pharmacological inhibition of NIK effectively mitigates gut inflammation in multiple preclinical models of colitis, including models with CD-like and UC-like features. These results underscore the therapeutic potential of NIK-smi as a promising intervention for different subtypes of IBD.

## Discussion

This study establishes the noncanonical NF-κB pathway, driven by NIK, as an essential regulator of gut inflammation, integrating its roles across epithelial, myeloid, and lymphoid compartments. By leveraging cell-type-specific and systemic models, we demonstrated how NIK orchestrates key processes in IBD pathogenesis, including immune cell recruitment, pro-inflammatory signaling, and epithelial-immune crosstalk.

At the epithelial level, the RANK-NIK axis is critical for M-cell differentiation and maintenance. Dysregulated M-cells, which are elevated in IBD tissues, produce CCL20, and are therefore poised to drive the recruitment of CCR6-expressing inflammatory cells, including Th17 cells and ILC3s, both of which are strongly associated with IBD (44, 45). The genetic association of *CCL20* and *CCR6* with IBD further underscores the importance of this chemokine axis in disease pathogenesis (46, 47).

In the immune cell compartment, NIK activity in CD11c+ DCs is associated with increased production of chemokines such as CCL5 and CXCL1 and with enhanced monocyte and neutrophil recruitment, consistent with a role for NIK in amplifying innate immune responses. Similarly, in the adaptive immune compartment, NIK drives OX40-mediated T cell activation, promoting a pro-inflammatory Th1 response while suppressing Tregs. These findings highlight the compartmentalized yet interconnected role of NIK in shaping IBD-associated inflammation.

Systemic NIK inhibition, achieved through both genetic and pharmacologic approaches, consistently attenuated inflammation in preclinical models. The dose-dependent efficacy observed with the small molecule NIK inhibitor, supported by a PK/PD model and pathway biomarker modulation, further validates the compelling therapeutic potential of targeting NIK. However, because NIK regulates noncanonical NF-κB signaling in multiple immune and stromal compartments, systemic inhibition is also expected to exert broader immunosuppressive effects beyond the intestinal mucosa, and the observed efficacy likely reflects a combination of local and systemic immunomodulatory actions. By integrating findings across multiple cellular compartments and disease models, this work identifies NIK as a key regulator of the inflammatory cascade in IBD. These results provide a strong rationale for developing more efficacious (i.e., lower dose) NIK inhibitors as a therapeutic strategy for IBD. Future studies should explore the translational potential of NIK inhibitors, particularly in combination with existing therapies, to address the unmet needs in patients with IBD, while carefully considering potential safety liabilities.

From a translational perspective, the essential roles of NIK in B cell survival, germinal center formation, lymphoid organ integrity, and antimicrobial host defense raise important safety considerations for systemic NIK inhibition. Genetic ablation of NIK in adult mice disrupts mature B cell survival and antibody responses (26, 40), and NIK deficiency in humans is associated with immunodeficiency phenotypes (48). Therefore, while our data support a strong therapeutic rationale for targeting NIK in IBD, they also suggest that careful dose selection, monitoring of humoral and cellular immunity, and possibly tissue- or cell type-selective targeting strategies may be required to mitigate immunological liabilities such as increased susceptibility to infections or impaired vaccine responses.

## Data Availability

The original contributions presented in the study are included in the article/**Supplementary Material**. Further inquiries can be directed to the corresponding author/s.

## Supplementary material

Supplementary Figure 1 M-cells in colon patches are regulated by the RANK-NIK pathway. **(A)** RNAscope *in situ* hybridization (ISH) of *Ccl20* in the colon patches of NIK.F/F and NIK.F/F;Villin.Cre mice. **(B, C)** RNAscope ISH of *Gp2* **(B)** and quantitative PCR analysis of *Gp2* and *Ccl20* expression **(C)** in colon patches of wild-type mice after 1 week of anti-RANKL antibody treatment. **(D)** Assessment of colitis severity in NIK.F/F and NIK.F/F;Villin.Cre.ERT2 mice in the *Citrobacter rodentium* infection model, including colon length and colon density (colon weight-to-length ratio). Data are based on analyses of 8 to 9 mice per group. Statistical significance is presented as mean ± SD. Unpaired t-tests were performed with Welch’s correction applied where appropriate. Statistical significance is indicated as follows: *, P < 0.05; **, P < 0.01; ***, P < 0.001; ****, P < 0.0001.

Supplementary Figure 2 Pseudobulk expression of M-cell marker genes across epithelial cell types. **(A)** Pseudobulk expression of M-cell marker genes in the ileal epithelium from Thomas et al., 2024 (PMID: 39438660). **(B)** Pseudobulk expression of M-cell marker genes in the colonic epithelium from Thomas et al., 2024 (PMID: 39438660). **(C)** Pseudobulk expression of M-cell marker genes in the colonic epithelium from Smillie et al., 2019 (PMID: 31348891).

Supplementary Figure 3 Pharmacologic inhibition of NIK demonstrated efficacy in T cell transfer colitis model. **(A)** Experimental schematic for the adoptive T cell transfer-induced colitis model. CD45RB^hi^ naïve CD4+ T cells from WT C57BL/6 donor mice were transferred into Rag1-/- recipient mice, with vehicle or NIK-smi treated twice a day **(BID)** starting from D0. **(B, C)** Assessment of colitis severity in recipient mice, including colon length **(B)** and colon density (colon weight-to-length ratio) **(C)**. **(D)** Cytokine profiles of colon tissues, including IL-6, IL-23a, IFN-γ, TNF and soluble CD40L (sCD40L). Data are based on analyses of 9 or 10 mice per group. Statistical significance is presented as mean ± SD. Mann-Whitney *U* tests were applied where appropriate. Statistical significance is indicated as follows: *, P < 0.05; **, P < 0.01; ***, P < 0.001; ****, P < 0.0001.

Supplementary Figure 4 Pharmacologic inhibition of NIK demonstrated efficacy in DSS-induced DSS colitis model. **(A)** Experimental schematic for the DSS-induced colitis model. WT C57BL/6 were fed on DSS water from Day 0 to Day 7 first to induce colitis, and then treated with vehicle or NIK-smi twice a day (BID) starting from Day 8. **(B, C)** Assessment of colitis severity in the two groups, including body weight changes **(B)**, colon length and colon density (colon weight-to-length ratio) **(C)**. **(D)** Quantification of total cell numbers of CD4_+_ T cells and effector Th17 or Th1 cells in the colon lamina propria by flow cytometry analysis (gating strategies in **Supplementary Figure 5B**). **(E)** Cytokine profiles of colon tissues, including IL-1β, IL-6, and CCL5. Data are based on analyses of 14 or 15 mice per group. Statistical significance is presented as mean ± SD. RM two-way ANOVA (B) or Mann-Whitney *U* tests were applied where appropriate. Statistical significance is indicated as follows: *, P < 0.05; **, P < 0.01; ***, P < 0.001; ****, P < 0.0001.

Supplementary Figure 5 Gating strategies employed for flow cytometry analysis. **(A)** Gating strategies for neutrophils (alive CD45^+^CD11b^+^Siglec-F^-^Ly6G^+^) and macrophages (alive CD45^+^CD11b^+^Ly6G^-^Siglec-F^-^CD64^+^). **(B)** Gating strategies for CD19^+^ B cells (alive CD45^+^TCRβ^-^CD19^+^), Foxp3^+^ Tregs (alive CD45^+^CD19^-^CD4^+^CD8a^-^TCRβ^+^Foxp3^+^), effector Th1 cells (alive CD45^+^CD19^-^CD4^+^CD8a^-^TCRβ^+^Foxp3^-^T-bet/IFNγ^+^), and effector Th17 cells (alive CD45^+^CD19^-^CD4^+^CD8a^-^TCRβ^+^Foxp3^-^RORγt/IL17A^+^). **(C)** Gating strategies for IL-22–expressing ILC3-like cells (alive CD45^+^CD90^+^Lineage^-^IL-22^+^).
