# Supplementary figures and images for: Noncanonical NF-κB pathway driven inflammation across multiple cellular compartments identifies NIK as a therapeutic target for inflammatory bowel disease
Source: Front Immunol. 2026 Jun 9;17:1825442. doi: 10.3389/fimmu.2026.1825442 (PMC13288176; doi:10.3389/fimmu.2026.1825442)

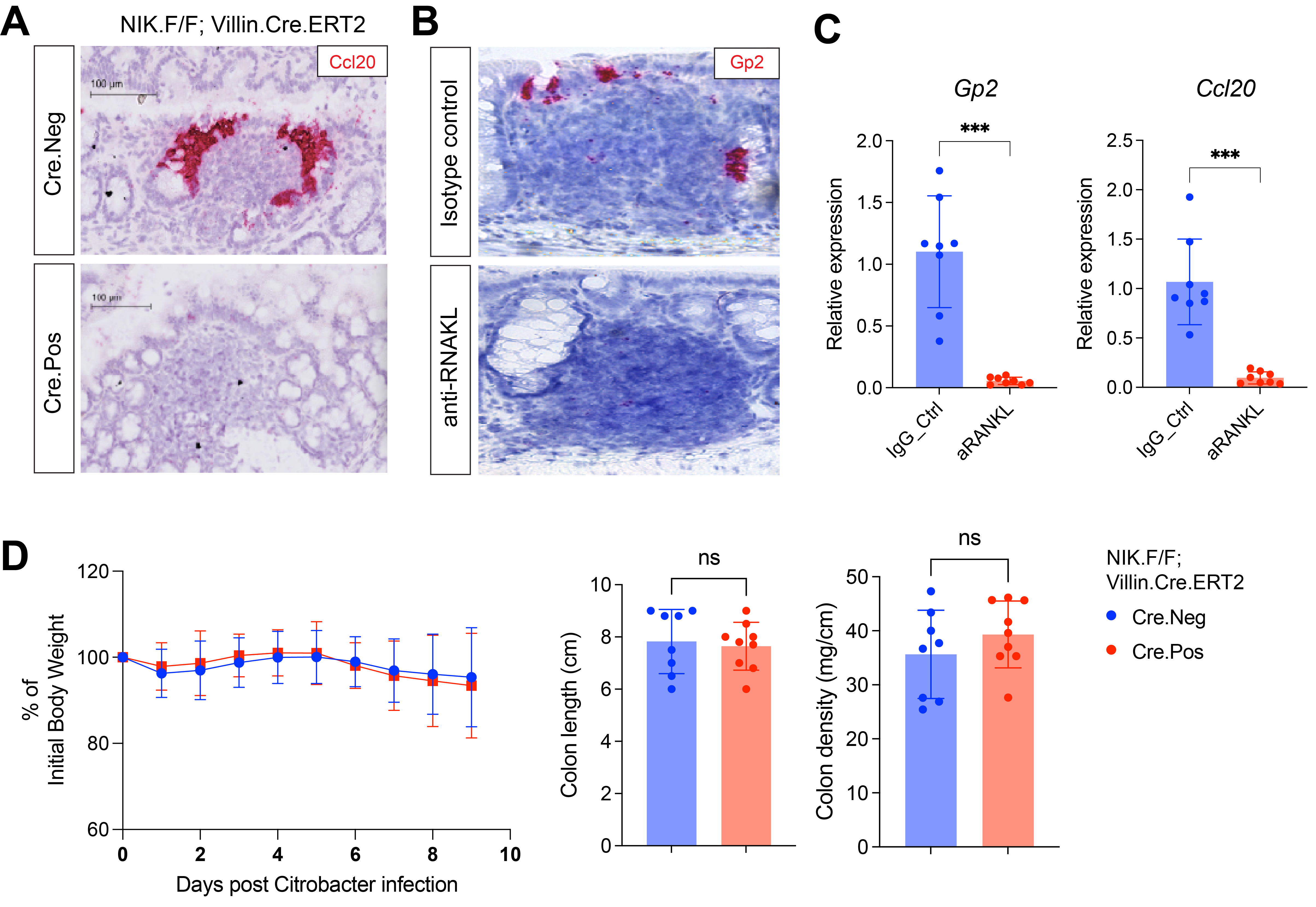

Supplement: Supplementary Figure 1 — M-cells in colon patches are regulated by the RANK-NIK pathway. (A) RNAscope in situ hybridization (ISH) of Ccl20 in the colon patches of NIK.F/F and NIK.F/F;Villin.Cre mice. (B, C) RNAscope ISH of Gp2 (B) and quantitative PCR analysis of Gp2 and Ccl20 expression (C) in colon patches of wild-type mice after 1 week of anti-RANKL antibody treatment. (D) Assessment of colitis severity in NIK.F/F and NIK.F/F;Villin.Cre.ERT2 mice in the Citrobacter rodentium infection model, including colon length and colon density (colon weight-to-length ratio). Data are based on analyses of 8 to 9 mice per group. Statistical significance is presented as mean ± SD. Unpaired t-tests were performed with Welch’s correction applied where appropriate. Statistical significance is indicated as follows: *, P < 0.05; **, P < 0.01; ***, P < 0.001; ****, P < 0.0001. [file Image1.png]

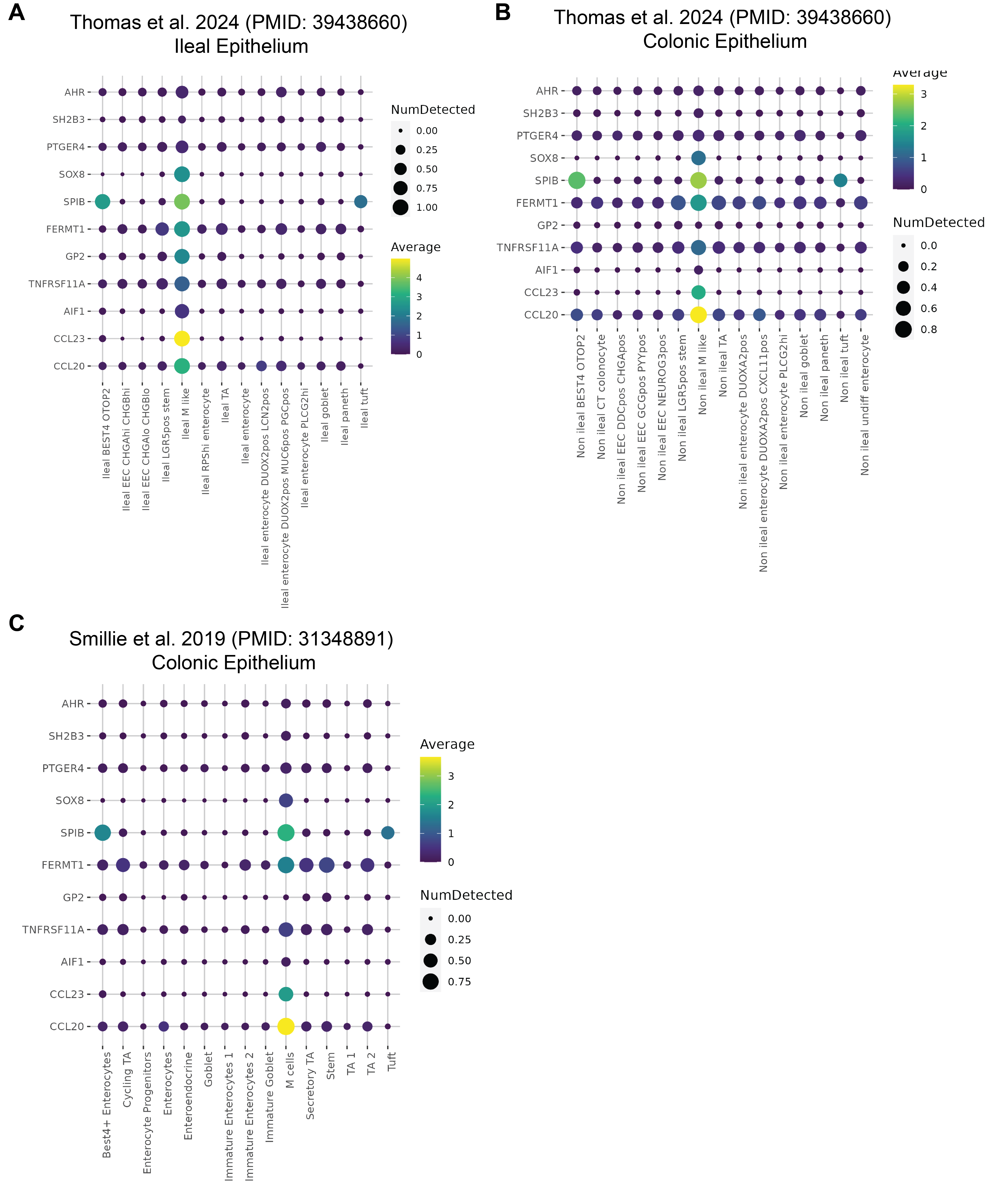

Supplement: Supplementary Figure 2 — Pseudobulk expression of M-cell marker genes across epithelial cell types. (A) Pseudobulk expression of M-cell marker genes in the ileal epithelium from Thomas et al., 2024 (PMID: 39438660). (B) Pseudobulk expression of M-cell marker genes in the colonic epithelium from Thomas et al., 2024 (PMID: 39438660). (C) Pseudobulk expression of M-cell marker genes in the colonic epithelium from Smillie et al., 2019 (PMID: 31348891). [file Image2.png]

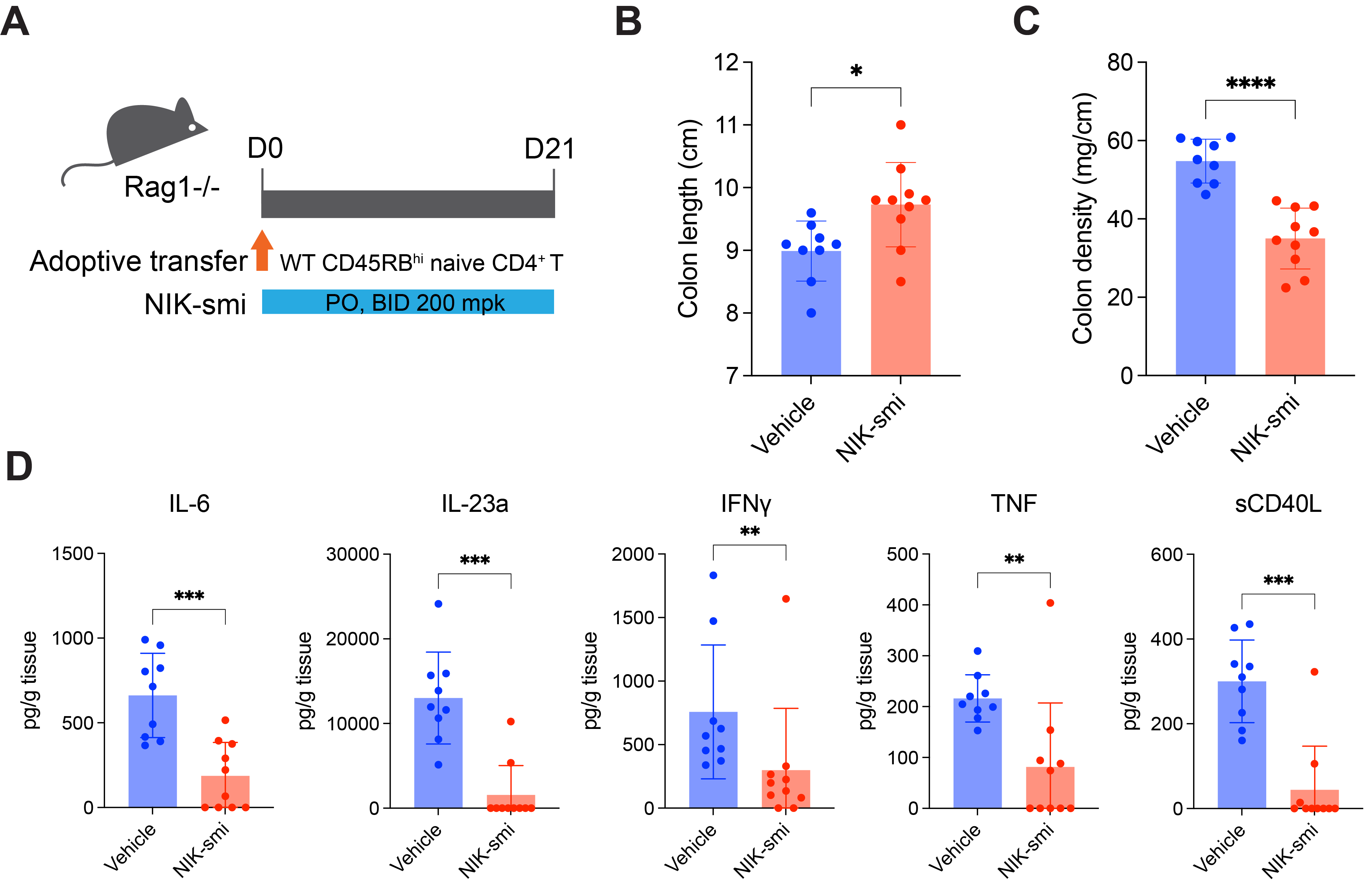

Supplement: Supplementary Figure 3 — Pharmacologic inhibition of NIK demonstrated efficacy in T cell transfer colitis model. (A) Experimental schematic for the adoptive T cell transfer-induced colitis model. CD45RBhi naïve CD4+ T cells from WT C57BL/6 donor mice were transferred into Rag1-/- recipient mice, with vehicle or NIK-smi treated twice a day (BID) starting from D0. (B, C) Assessment of colitis severity in recipient mice, including colon length (B) and colon density (colon weight-to-length ratio) (C). (D) Cytokine profiles of colon tissues, including IL-6, IL-23a, IFN-γ, TNF and soluble CD40L (sCD40L). Data are based on analyses of 9 or 10 mice per group. Statistical significance is presented as mean ± SD. Mann-Whitney U tests were applied where appropriate. Statistical significance is indicated as follows: *, P < 0.05; **, P < 0.01; ***, P < 0.001; ****, P < 0.0001. [file Image3.png]

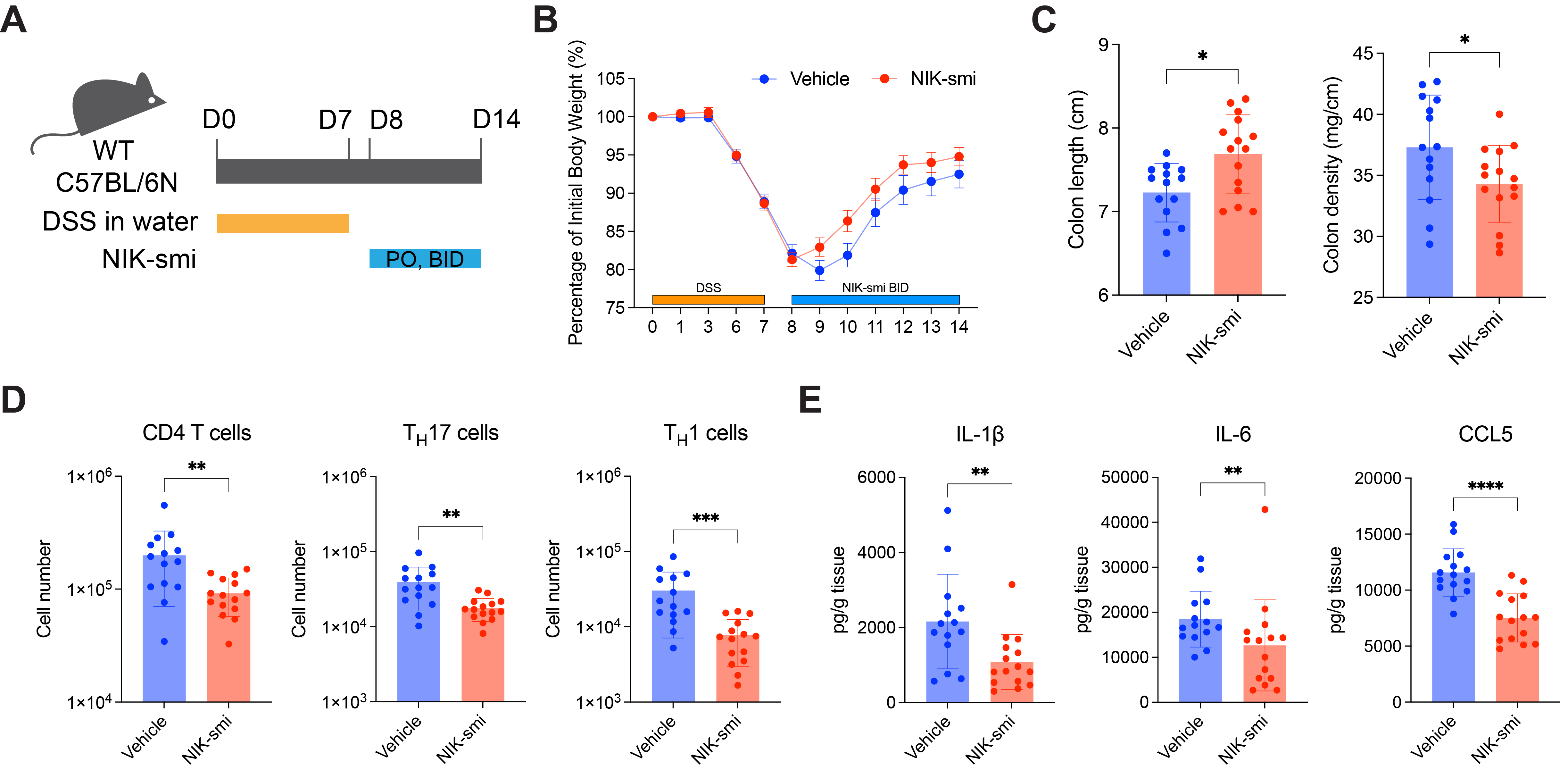

Supplement: Supplementary Figure 4 — Pharmacologic inhibition of NIK demonstrated efficacy in DSS-induced DSS colitis model. (A) Experimental schematic for the DSS-induced colitis model. WT C57BL/6 were fed on DSS water from Day 0 to Day 7 first to induce colitis, and then treated with vehicle or NIK-smi twice a day (BID) starting from Day 8. (B, C) Assessment of colitis severity in the two groups, including body weight changes (B), colon length and colon density (colon weight-to-length ratio) (C). (D) Quantification of total cell numbers of CD4+ T cells and effector Th17 or Th1 cells in the colon lamina propria by flow cytometry analysis (gating strategies in Supplementary Figure 5B). (E) Cytokine profiles of colon tissues, including IL-1β, IL-6, and CCL5. Data are based on analyses of 14 or 15 mice per group. Statistical significance is presented as mean ± SD. RM two-way ANOVA (B) or Mann-Whitney U tests were applied where appropriate. Statistical significance is indicated as follows: *, P < 0.05; **, P < 0.01; ***, P < 0.001; ****, P < 0.0001. [file Image4.png]

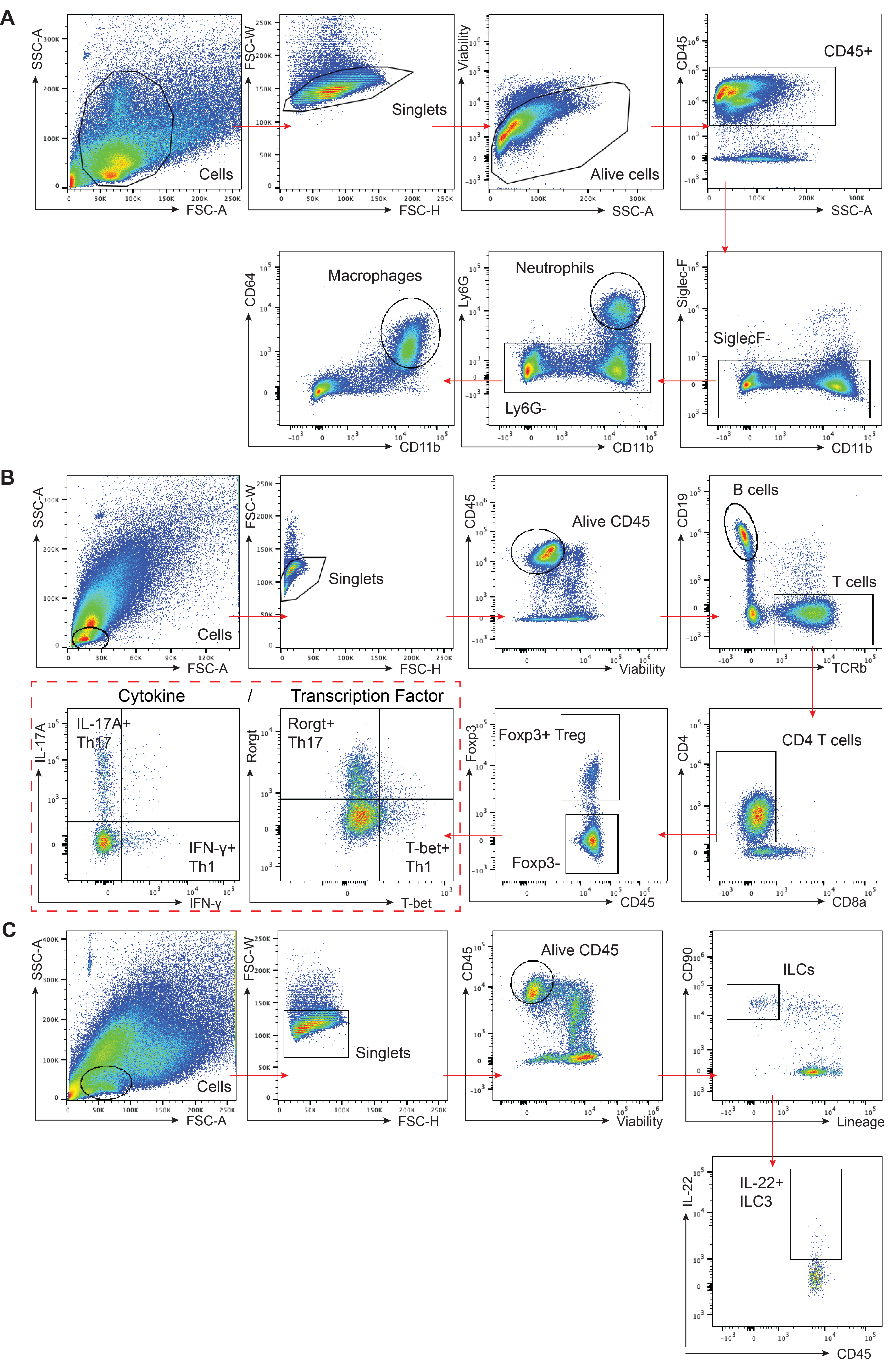

Supplement: Supplementary Figure 5 — Gating strategies employed for flow cytometry analysis. (A) Gating strategies for neutrophils (alive CD45+CD11b+Siglec-F-Ly6G+) and macrophages (alive CD45+CD11b+Ly6G-Siglec-F-CD64+). (B) Gating strategies for CD19+ B cells (alive CD45+TCRβ-CD19+), Foxp3+ Tregs (alive CD45+CD19-CD4+CD8a-TCRβ+Foxp3+), effector Th1 cells (alive CD45+CD19-CD4+CD8a-TCRβ+Foxp3-T-bet/IFNγ+), and effector Th17 cells (alive CD45+CD19-CD4+CD8a-TCRβ+Foxp3-RORγt/IL17A+). (C) Gating strategies for IL-22–expressing ILC3-like cells (alive CD45+CD90+Lineage-IL-22+). [file Image5.png]
